# A pH‐Responsive Biomimetic Antioxidant Nanoplatform with Dual Renal Targeting for Synergistic Therapy of Acute Kidney Injury

**DOI:** 10.1002/advs.202515664

**Published:** 2025-11-06

**Authors:** Shichao Zhang, Yuhan Xie, Longchao Zhang, Yuanjiong Qi, Quan Liao, Chenglong Xu, Shushuai Yang, Haiwen Zhou, Qidan Tan, Shiyong Qi

**Affiliations:** ^1^ Department of Urology Tianjin Institute of Urology The Second Hospital of Tianjin Medical University Tianjin 300211 China; ^2^ Intensive care unit Institute of Infectious Diseases The Second Hospital of Tianjin Medical University Tianjin 300211 China

**Keywords:** acute kidney injury, biomimetic nanoparticles, macrophage membrane camouflage, pH‐responsive drug release, reactive oxygen species scavenging

## Abstract

Acute kidney injury (AKI) represents a critical clinical condition marked by abrupt deterioration of renal function, primarily driven by oxidative stress, inflammation, and apoptosis. However, effective targeted therapies remain limited. Here, a smart, biomimetic nanoplatform (CeAst@MK) that synergistically addresses oxidative and inflammatory injury in AKI is reported. CeAst nanoparticles are formed via coordination between Ce^3^⁺ ions and astragalin (Ast), a natural flavonoid with intrinsic ROS‐scavenging and anti‐inflammatory properties. To enhance immune evasion and renal targeting specificity, CeAst is cloaked with macrophage membranes (MCM) and modified with a kidney‐targeting peptide (KTP), yielding the final CeAst@MK system. The platform exhibits pH‐responsive release in the acidic microenvironment of injured renal tissues, enabling precise and rapid therapeutic delivery. In both LPS‐ and ischemia reperfusion‐induced AKI models, CeAst@MK significantly improves renal function, suppresses proinflammatory cytokines, and promotes M2 macrophage polarization. Mechanistically, it modulates PI3K/Akt and NF‐κB pathways, achieving dual antioxidative and anti‐inflammatory effects. This study presents a translationally promising nanotherapeutic system integrating natural antioxidants, biomimetic camouflage, and tissue‐specific delivery, offering an effective and precise strategy for AKI intervention.

## Graphical abstract

A biomimetic nanoplatform (CeAst@MK) is engineered for precise therapy of acute kidney injury by integrating cerium‐based antioxidation, natural flavonoid astragalin, and macrophage membrane camouflage. Featuring kidney‐targeted and pH‐responsive release, CeAst@MK scavenges ROS, suppresses inflammation, and restores renal function via modulation of PI3K/Akt and NF‐κB pathways in LPS‐ and IRI‐induced models.

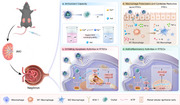

## 1 Introduction

Acute kidney injury (AKI) is a clinically prevalent and severe pathological condition characterized by a rapid decline in renal function, often triggered by ischemia‐reperfusion injury (IRI), sepsis, nephrotoxic drugs, or metabolic disturbances. Globally, over 13 million individuals suffer from AKI annually, with mortality rates exceeding 50% in severe cases.^[^ ^1^ ^]^ The pathogenesis of AKI is multifactorial, involving tissue hypoxia, inflammatory responses, excessive ROS accumulation, mitochondrial dysfunction, and tubular epithelial cell apoptosis.^[^ ^2^ ^]^ Notably, ischemia reperfusion‐induced hypoxia rapidly activates pathways such as xanthine oxidase (XO), resulting in aberrant accumulation of superoxide anions (•O_2_^−^) and hydrogen peroxide (H_2_O_2_), thereby amplifying oxidative stress and local inflammation, which accelerates renal tissue injury.^[^ ^3^, ^4^, ^5^ ^]^ Despite advances in supportive care, effective targeted therapeutic interventions for AKI remain elusive.^[^ ^6^ ^]^

In this context, the development of novel therapeutics with potent ROS scavenging capabilities has become a critical direction for AKI management. Nanomedicine has recently emerged as a promising approach to mitigate ROS‐mediated tissue damage. Cerium‐based nanomaterials, owing to the reversible redox cycling between Ce^3^⁺ and Ce⁴⁺, exhibit superoxide dismutase (SOD) and catalase (CAT) mimetic activities, enabling efficient ROS neutralization and attenuation of oxidative stress within inflamed tissues.^[^ ^7^, ^8^, ^9^ ^]^ Moreover, these ceria nanomaterials can catalyze oxygen generation in ROS‐rich pathological microenvironments, thereby alleviating localized hypoxia and disrupting the vicious cycle of “hypoxia‐inflammation‐injury.”^[^ ^10^ ^]^ However, current ceria‐based therapeutic systems face challenges, including low catalytic efficiency, complex synthesis procedures, and limitations in biosafety and scalability, underscoring the urgent need for facile, efficient, and biocompatible antioxidative nanoplatforms.

Flavonoids have emerged as a class of bioactive molecules with potent antioxidant and anti‐inflammatory activities, positioning them as promising therapeutic candidates for oxidative stress‐associated disorders such as AKI.^[^ ^11^ ^]^ Among them, astragalin (Ast), a naturally occurring flavonoid, has attracted growing interest in AKI therapy owing to its rich phenolic hydroxyl content, which endows it with strong ROS scavenging capability and the ability to modulate key inflammatory signaling pathways.^[^ ^12^ ^]^ Moreover, Ast mediates macrophage phenotype switching from pro‐inflammatory (M1) to anti‐inflammatory (M2) states, effectively modulating cytokine‐mediated inflammatory responses while enhancing tissue regeneration and microenvironmental stabilization. However, the clinical translation of Ast remains severely hindered by its poor aqueous solubility, rapid systemic metabolism, and limited bioavailability.^[^ ^13^, ^14^ ^]^ To address these pharmacokinetic limitations, we rationally designed a cerium–astragalin coordination nanocomplex (CeAst), which leverages the intrinsic redox‐switching behavior of Ce^3^⁺/Ce⁴⁺ to synergistically boost ROS scavenging efficiency, enhance drug stability, improve loading capacity, and endow catalytic antioxidative functionality.

Furthermore, biomimetic nanotechnology has demonstrated substantial potential in enhancing nanoparticle targeting, biocompatibility, and in vivo stability. Cell membrane coating strategies, particularly those employing macrophage cell membranes (MCM), endow nanoparticles with “self‐recognition” properties, facilitating immune evasion and selective accumulation within inflammatory microenvironments.^[^ ^15^, ^16^ ^]^ MCM surfaces naturally express adhesion molecules and chemokine receptors that augment inflammatory site targeting and promote trans‐endothelial migration. Nonetheless, limitations such as insufficient targeting specificity, challenges in membrane sourcing, and protein stability constrain their broader application in precise therapies.^[^ ^17^ ^]^

To address these limitations and further improve systemic delivery and targeting efficiency, we developed a biomimetic nanoplatform by coating CeAst with MCM, yielding the CeAst@MK system. Additionally, we functionalized the nanoparticle surface with a kidney‐targeting peptide (KTP), capable of specifically recognizing kidney injury molecule‐1 (KIM‐1), which is markedly upregulated in early AKI tubular damage, thereby achieving precise localization to injured renal tissue and significantly enhancing accumulation and therapeutic efficacy at AKI sites.^[^ ^18^, ^19^ ^]^

**Scheme 1:**
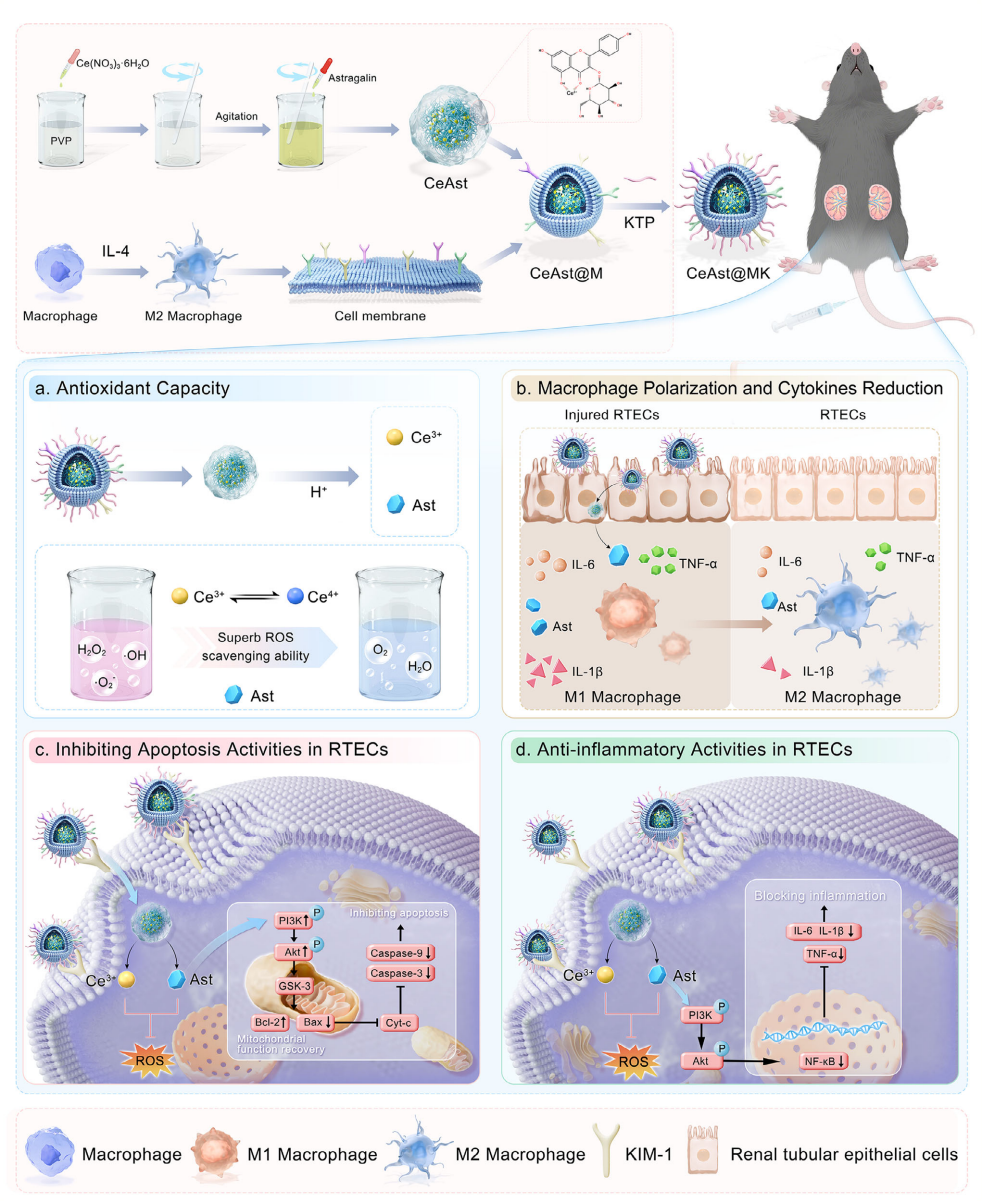
Schematic illustration of CeAst@MK for targeted therapy of AKI, featuring ROS scavenging, antioxidant, anti‐apoptotic, and anti‐inflammatory properties.

Building upon this rationale, we constructed the novel biomimetic antioxidative nanoplatform CeAst@MK to achieve precise AKI lesion targeting coupled with pH‐responsive drug release for synergistic antioxidative and anti‐inflammatory therapy. As illustrated in **Scheme** **1**, this system integrates four synergistic advantages: 1) Dual targeting conferred by MCM and KTP modification enables selective homing to inflammatory regions and injured renal tissue; 2) pH‐triggered release in the acidic inflammatory microenvironment enhances the cooperative ROS scavenging capabilities of Ce^3^⁺ and Ast, effectively alleviating oxidative stress and tissue hypoxia; 3) The phenotypic shift from M1 to M2 macrophages mediates significant reductions in pro‐inflammatory cytokine production; 4) Modulation of the PI3K/Akt and NF‐κB signaling pathways facilitates coordinated antioxidative and anti‐inflammatory therapeutic effects. Collectively, this study presents a promising biomimetic nanotherapeutic strategy, providing a robust theoretical foundation and practical avenue for the precise treatment and clinical translation of AKI and related inflammatory kidney diseases.

## 2 Results and Discussion

### 2.1 Network Pharmacology Investigation of Ast for AKI Treatment

This research implemented an integrative network pharmacology strategy to systematically investigate the active components and therapeutic mechanisms of Eucommia ulmoides against AKI. Initial screening of 42 components from the TCMSP database using drug‐likeness (DL > 40%) and molecular weight (MW < 600) criteria yielded 26 potential active compounds. Target prediction via SwissTargetPrediction identified 386 unique targets, while GeneCards provided 1000 AKI‐related targets, with Venn analysis revealing 83 overlapping targets (Figure S1A, Supporting Information). To elucidate the potential mechanisms of Eucommia ulmoides‐derived compounds in AKI therapy, a protein–protein interaction (PPI) network was constructed based on AKI‐related targets, and topological analysis using the Centiscape plugin identified 18 hub genes, including TNF, IL2, AKT1, and EGFR (Figure S1B, Supporting Information). Functional enrichment analysis via DAVID revealed significant involvement of these genes in AKI‐associated biological processes and signaling pathways, notably the inflammatory response, NF‐κB signaling, and PI3K‐Akt pathway (Figure S1D–G, Supporting Information). Integration of component–target–pathway networks prioritized 15 bioactive candidates, with Ast emerging as a key therapeutic compound (Figure S1C, Supporting Information). Molecular docking demonstrated a strong binding affinity between Ast and AKT1, with a calculated binding energy of −9.0 kcal mol^−1^ (Figure S2, Supporting Information), suggesting a high probability of direct interaction. Together, these results highlight the therapeutic potential of Ast and provide a mechanistic basis for its application in AKI treatment.

### 2.2 Preparation and Characterization of CeAst@MK

Building upon the identification of Ast as a promising therapeutic candidate, we engineered a biomimetic nano‐antioxidant platform, CeAst@MK, designed to enhance pharmacological efficacy through nanomaterial‐mediated delivery. This platform features a core nanostructure formed via the coordination‐driven self‐assembly of Ast with cerium ions (CeAst), subsequently cloaked with MCM and functionalized with a KTP to confer immune evasion and renal specificity (Scheme 1). The CeAst core was synthesized through a robust coordination process wherein cerium nitrate (Ce(NO_3_)_3_·6H_2_O) interacts with Ast in ethanol under the stabilizing influence of polyvinylpyrrolidone (PVP). To endow the construct with inflammation‐homing and immune camouflage properties, MCM derived from RAW264.7 cells was co‐extruded with CeAst, followed by surface conjugation of KTP, culminating in the formation of the fully functionalized CeAst@MK nanoparticles.

**Figure 1:**
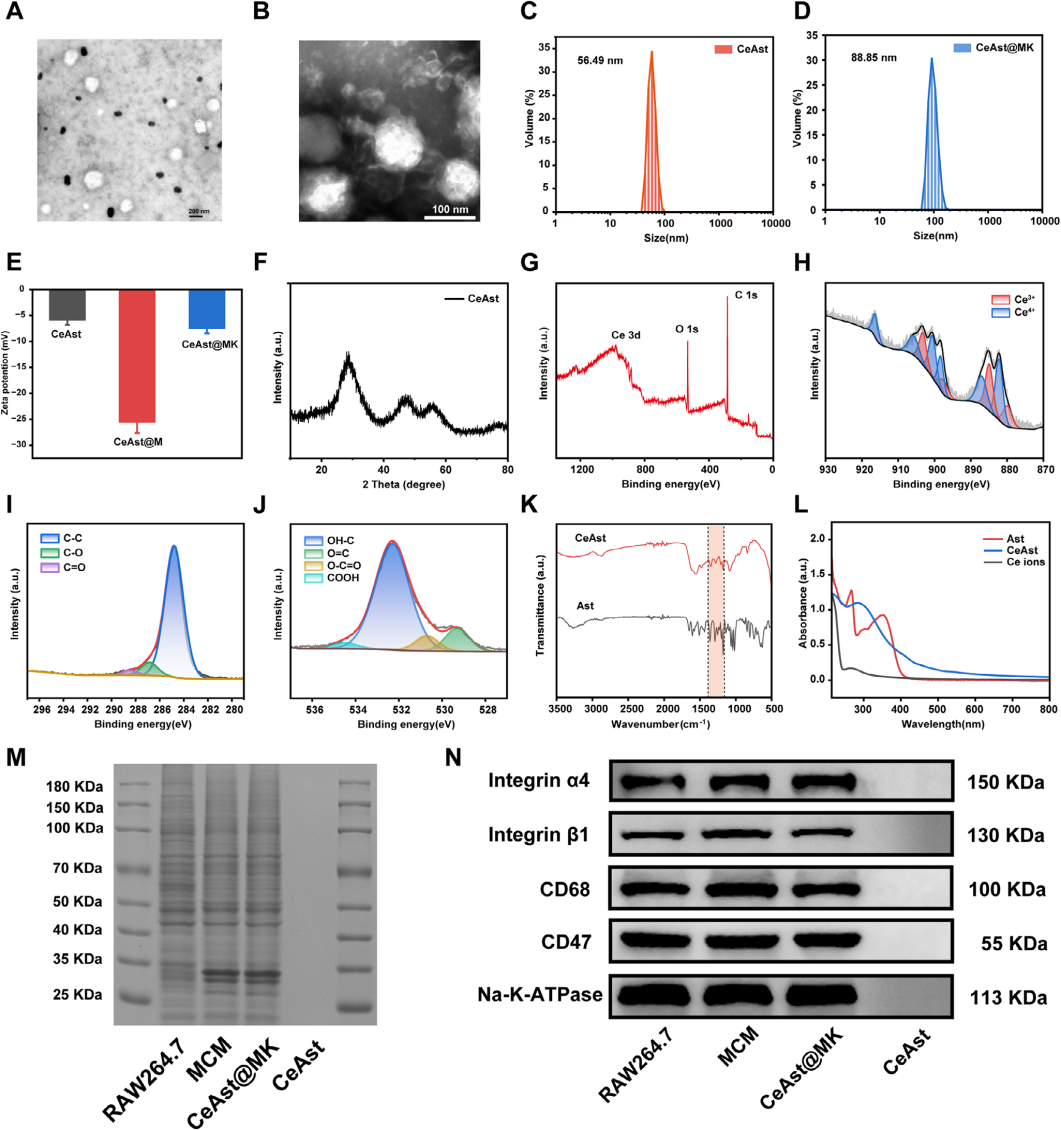
Synthesis and Characterization of CeAst@MK. A,B) TEM images of CeAst and CeAst@MK. C,D) Size distribution of as‐prepared CeAst and CeAst@MK. E) Zeta potentials of CeAst, CeAst@M, and CeAst@MK. F) XRD pattern of CeAst@MK. G–J) XPS survey spectrum and high‐resolution scans of Ce 3d, C 1s, and O 1s peaks for CeAst@MK. K) FT‐IR spectra of Ast and CeAst. L) UV‐vis absorption spectra of Ast and CeAst. M) Protein profiles of RAW 264.7 cells, MCM, CeAst@MK, and CeAst analyzed by SDS‐PAGE. N) Membrane surficial proteins on RAW 264.7 cells, MCM, CeAst@MK, and CeAst.

Transmission electron microscopy (TEM) and dynamic light scattering (DLS) analyses indicated that CeAst exhibited a uniform spherical morphology with an average diameter of 56.49 nm (**Figure** **1**A,C). Upon membrane coating, CeAst@MK retained its spherical shape but increased in size to 88.85 nm (Figure 1B,D), consistent with the estimated membrane thickness of 20–30 nm and in line with prior reports.^[^ ^20^, ^21^, ^22^ ^]^ Additionally, zeta potential measurements revealed a shift from −5.83 mV (CeAst) to −25.51 mV (CeAst@M) (Figure 1E), supporting successful membrane encapsulation. However, the zeta potential of CeAst@MK further shifted to −7.42 mV, providing additional evidence for the successful modification with KTP. X‐ray diffraction (XRD) analysis revealed a broad amorphous profile for CeAst@MK, with no crystalline peaks detected, indicating a high purity, non‐crystalline cerium oxide structure (Figure 1F). X‐ray photoelectron spectroscopy (XPS) revealed characteristic peaks of Ce (899.07 eV), O (532.17 eV), and C (285.18 eV) (Figure 1G–J), confirming elemental composition. High‐resolution Ce 3d spectra revealed the coexistence of Ce^3^⁺ and Ce⁴⁺, with a Ce^3^⁺/Ce⁴⁺ molar ratio of 0.86, indicating a mixed valence state conducive to dual enzyme mimetic activities, with Ce^3^⁺ conferring SOD‐like properties and Ce⁴⁺ enhancing CAT‐like activity.^[^ ^23^ ^]^ The successful coordination between Ce^3^⁺ and Ast within the CeAst@MK nanocomposite was unequivocally confirmed through comprehensive spectroscopic characterization. Fourier transform infrared (FT‐IR) spectroscopy revealed a marked attenuation of the C–OH stretching vibration near 1300 cm^−1^, indicative of strong chelation between Ce^3^⁺ ions and the phenolic hydroxyl groups of Ast (Figure 1K). Complementarily, UV–vis absorption spectroscopy demonstrated a pronounced red shift in CeAst@MK relative to free Ast (345 nm), reflecting perturbations in π‐conjugation arising from metal ligand complexation (Figure 1L).

DLS analysis further substantiated the colloidal robustness of CeAst and CeAst@MK, with a stable and uniform hydrodynamic diameter maintained in both aqueous media and DMEM at 37 °C for over seven days (Figure S3A,B, Supporting Information), underscoring their exceptional physiological stability. We conducted in vitro drug release experiments under physiological (pH 7.4), mildly acidic (pH 6.5), and acidic (pH 5.5) conditions (Figure S4, Supporting Information). The results showed that CeAst@MK exhibited a markedly accelerated release of Ast in the acidic environment, thereby confirming the pH‐responsive property of CeAst@MK.^[^ ^24^ ^]^ In parallel, previous studies have reported that in AKI models induced by IRI or sepsis, hypoxia‐driven glycolytic enhancement and lactate accumulation lead to significant acidification of the renal tubules, with cortical pH dropping to ≈6.5–6.8.^[^ ^25^, ^26^ ^]^ Taken together, these findings indicate that the pathological regions of AKI provide the requisite acidic microenvironment to trigger the responsive release of our nanoparticles.

Protein retention and membrane integrity upon cloaking were validated via SDS‐PAGE, which confirmed the preservation of membrane proteins on CeAst@MK comparable to purified MCM, in stark contrast to protein‐absent CeAst cores (Figure 1M). Western blot analyses corroborated the presence of critical functional membrane proteins, including integrin α4/β1 and the immune “self” marker CD47, on both MCM and CeAst@MK, but were notably absent from CeAst alone (Figure 1N). The CD47‐SIRP‐α axis is pivotal for evading phagocytic clearance by delivering a “don't eat me” signal.^[^ ^27^, ^28^ ^]^ Collectively, these results confirm that membrane coating preserves essential protein functionality, thereby endowing CeAst@MK with immune evasion and inflammation targeting capabilities. Altogether, these findings validate the successful fabrication of CeAst@MK with favorable dispersibility and bio‐functionality, laying a robust foundation for its prospective in vivo therapeutic applications.

### 2.3 ROS Scavenging Capability of CeAst@MK

**Figure 2:**
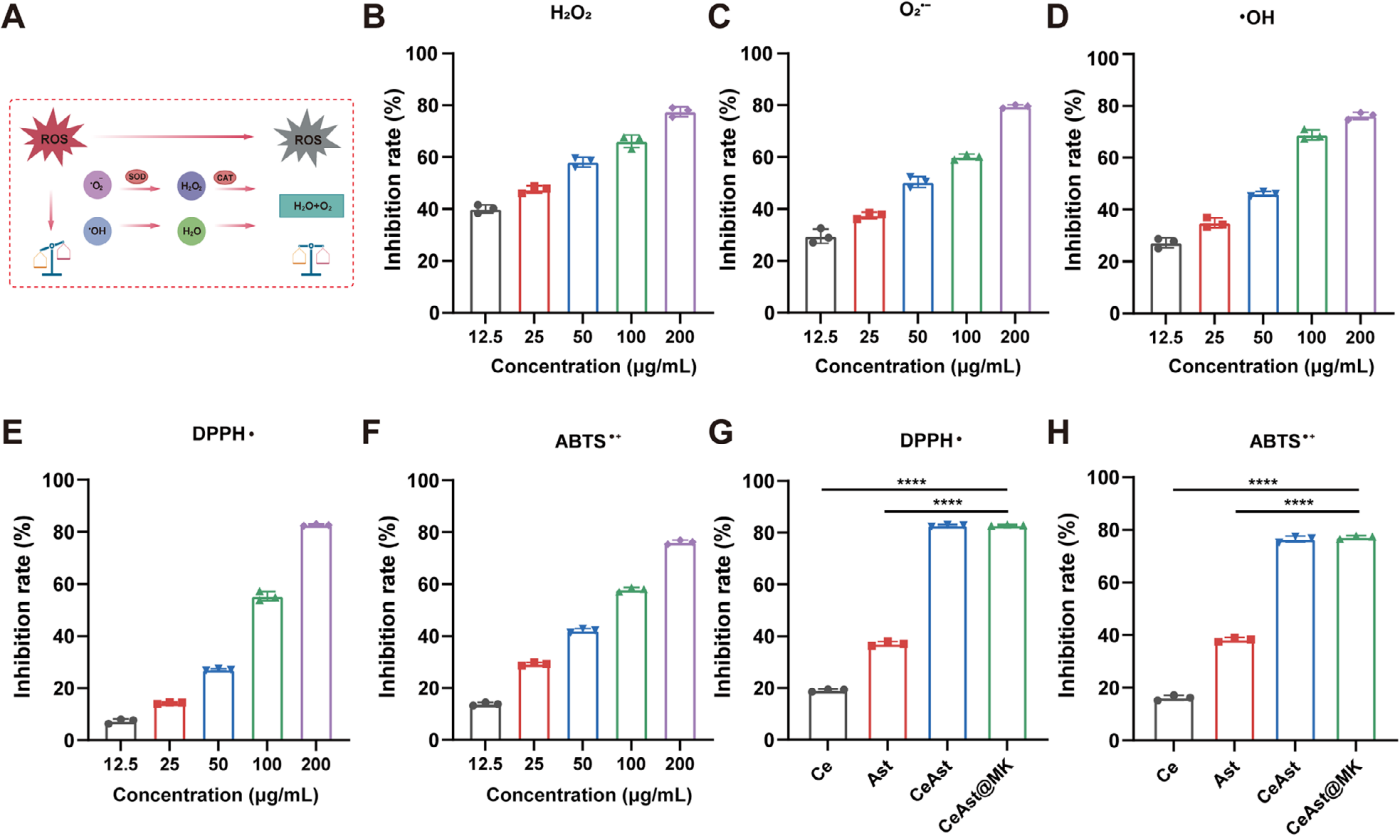
Antioxidant activity of CeAst@MK. A) Schematic illustration of antioxidative mechanisms of CeAst@MK. B–F) Concentration‐dependent scavenging activities of CeAst@MK against H_2_O_2_, O_2_^•−^, •OH, DPPH•, and ABTS^•+^ (n = 3). G,H) Comparative DPPH• and ABTS•⁺ scavenging efficiencies of CeAst@MK, CeAst, Ast, and Ce ions (*n* = 3). The data were presented as mean ± SD (*n* = 3). *p < 0.05, **p < 0.01, ***p < 0.001, ****p < 0.0001, ns = not significant, one‐way ANOVA.

Excessive ROS production is a key feature of AKI, initiating oxidative stress, activating inflammatory cascades, and damaging renal tubular epithelial cells and microvasculature, thereby aggravating renal dysfunction. Therefore, developing nanotherapeutics with potent ROS scavenging capabilities represents a promising strategy for halting AKI progression. To assess the antioxidant potential of the CeAst@MK nanoplatform, we examined its ability to neutralize three representative ROS species: hydrogen peroxide (H_2_O_2_), superoxide anions (O_2_^•−^), and hydroxyl radicals (•OH) (**Figure** **2**A). CeAst@MK exhibited dose‐dependent scavenging activity against all three ROS species (Figure 2B–D). At 200 µg·mL^−1^, CeAst@MK removed approx≈77.5% of H_2_O_2_ and 79.6% of O_2_^•−^, demonstrating CAT and SOD‐like activities, respectively. Moreover, it efficiently scavenged •OH radicals, with clearance increasing from 27.2% at 12.5 µg·mL^−1^ to 76.1% at 200 µg·mL^−1^ (Figure 2D). The overall antioxidant capacity of CeAst@MK was assessed using the 1,1‐diphenyl‐2‐picrylhydrazyl (DPPH^●^) assay, a widely adopted method for measuring the scavenging of stable free radicals. DPPH^●^ exhibits a characteristic violet color that diminishes upon reaction with antioxidants, providing a quantifiable measure of radical scavenging efficiency.^[^ ^29^ ^]^ CeAst@MK showed dose‐dependent DPPH^●^ clearance, achieving 7.29% at 12.5 µg mL^−1^ and reaching 82.68% at 200 µg mL^−1^ (Figure 2E), confirming its robust antioxidant capacity. Complementary analysis using the ABTS^•+^ assay further validated the broad‐spectrum scavenging ability of CeAst@MK. Upon oxidation, ABTS forms a stable blue–green radical cation (ABTS^•+^), which is reduced by antioxidants, resulting in decreased absorbance.^[^ ^30^ ^]^ CeAst@MK demonstrated potent ABTS^•+^ scavenging, with a clearance rate of 76.22% at 200 µg mL^−1^ (Figure 2F), reinforcing its wide‐ranging ROS neutralization efficacy.

To elucidate the synergistic contribution of the individual components, we compared the antioxidant performance of Ast, Ce^3^⁺ ions, CeAst, and the assembled CeAst@MK nanocomposite. Across all tested models, including DPPH^●^ and ABTS^•+^, CeAst@MK outperformed both Ast and Ce^3^⁺ alone (Figure 2G,H), highlighting the synergistic enhancement in ROS scavenging activity conferred by coordination‐driven self‐assembly. Together, these results underscore the strong and broad‐spectrum antioxidant activity of CeAst@MK, which combines efficient clearance of various ROS species with excellent stability and catalytic performance. This potent redox modulation capability provides a compelling basis for its application in ROS‐driven diseases such as AKI.

### 2.4 Immune Evasion and Renal Targeting of CeAst@MK

**Figure 3:**
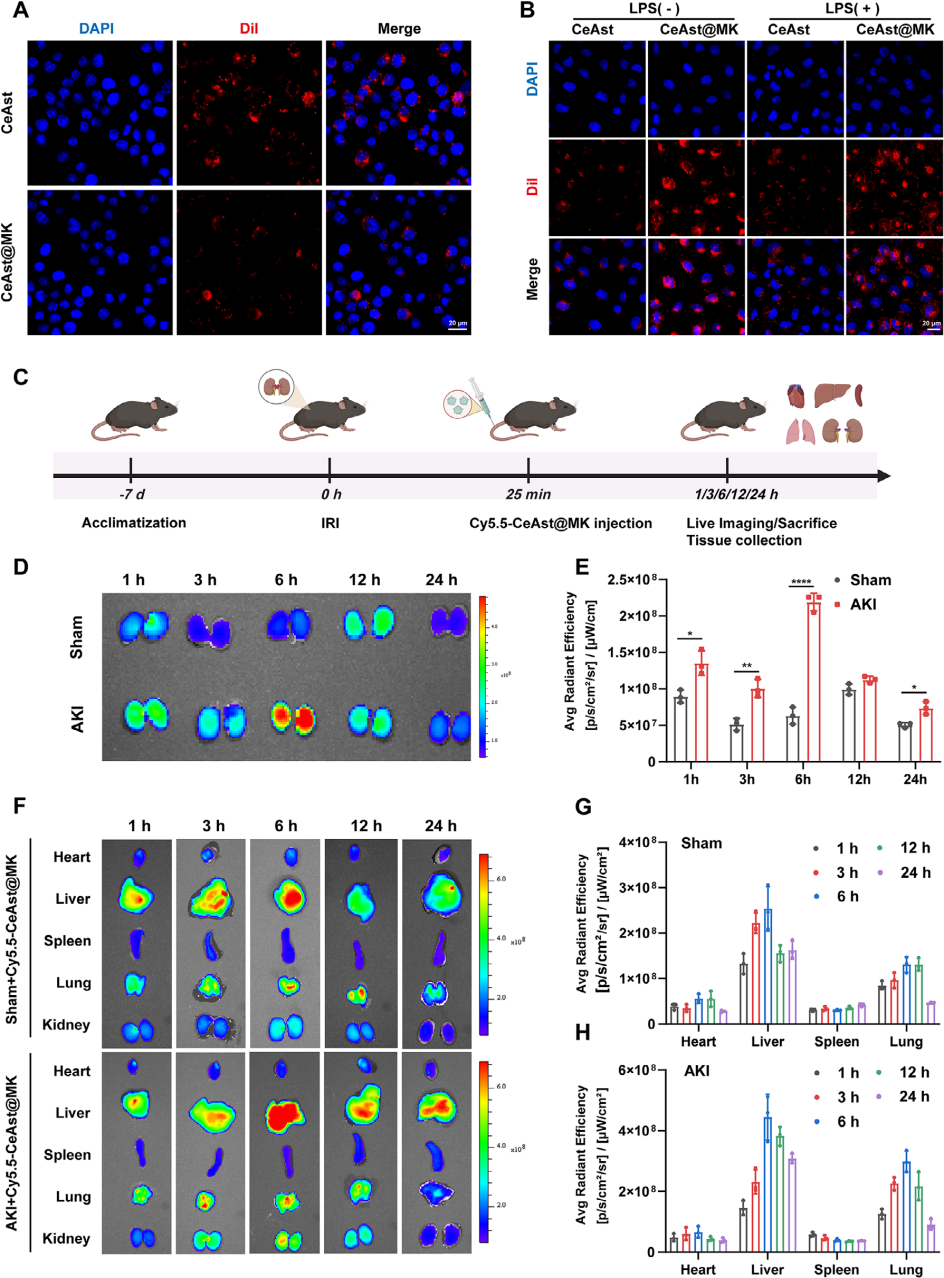
Immune evasion and renal targeting of CeAst@MK. A) CLSM images of RAW264.7 cells after incubation with DiI‐labeled CeAst or CeAst@MK for 6 h (n = 3). B) CLSM images of HK‐2 cells pretreated with LPS (1 µg mL^−1^, 1 h) or PBS, followed by incubation with DiI‐labeled CeAst or CeAst@MK for 6 h (n = 3). C) Schematic of the in vivo experimental design in a bilateral renal IRI model. D,E) In vivo fluorescence images and quantification of renal fluorescence intensity in mice administered Cy5.5‐CeAst@MK at indicated time points (*n* = 3). F–H) Biodistribution and semiquantitative fluorescence analysis of Cy5.5‐CeAst@MK in major organs (heart, liver, spleen, lung, and kidney) at different time intervals (*n* = 3). The data were shown as mean ± SD (*n* = 3). *p < 0.05, **p < 0.01, ***p < 0.001, ****p < 0.0001, ns indicates *p* > 0.05, by one‐way ANOVA.

To assess the immune evasion capability of CeAst@MK, confocal laser scanning microscopy (CLSM) was employed to visualize nanoparticle uptake in RAW264.7 cells (**Figure** **3**A). DiI‐labeled CeAst and CeAst@MK were co‐incubated with macrophages, and intracellular fluorescence was quantified. Compared to the uncoated CeAst group, CeAst@MK‐treated cells exhibited markedly reduced red fluorescence, indicating that macrophage membrane cloaking effectively suppressed phagocytic uptake, thereby endowing the nanoparticles with immune stealth functionality.

Building on these results, we further evaluated the inflammation‐targeting capacity of CeAst@MK. An in vitro inflammatory model was established by stimulating HK‐2 cells with 1 µg mL^−1^ LPS to mimic the AKI‐associated microenvironment.^[^ ^31^ ^]^ Following incubation with DiI‐labeled CeAst@MK, CLSM imaging revealed significantly enhanced fluorescence intensity in LPS‐treated cells compared to unstimulated controls (Figure 3B), demonstrating preferential accumulation in inflamed renal epithelium. Notably, CeAst@MK showed markedly stronger intracellular fluorescence than uncoated CeAst under identical conditions, suggesting that macrophage membrane modification substantially improved inflammation‐specific targeting. To investigate in vivo biodistribution, a murine IRI‐induced AKI model was established by clamping bilateral renal pedicles for 25 min, followed by tail vein injection of Cy5.5‐labeled CeAst@MK into sham and IRI groups (Figure 3C). Major organs were harvested at the indicated time points for ex vivo fluorescence imaging. As shown in Figure 3D–E, fluorescence intensity in IRI‐injured kidneys was significantly higher than in the sham group, confirming robust kidney targeting capacity in vivo.

The enhanced accumulation of CeAst@MK in injured renal tissues is attributed to its dual‐targeting design. The macrophage membrane enables homing to inflammatory sites via adhesion molecules and chemokine receptors, while the KTP selectively binds to KIM‐1, which is highly upregulated in damaged renal tubular epithelial cells during AKI. Together, these components synergistically enable precise lesion‐site accumulation. Moderate fluorescence was also observed in the liver and lungs of AKI mice, likely due to systemic inflammation‐induced endothelial barrier disruption and increased hepatic vascular permeability. Importantly, CeAst@MK exhibited prolonged renal retention, which is beneficial for sustained therapeutic effects and reduced systemic toxicity. As shown in Figure 3F–H, renal fluorescence intensity gradually increased, peaking at 6 h post‐injection, and remained detectable for up to 12 h. Fluorescence signals in other major organs progressively declined over time, indicating favorable in vivo biodegradability and clearance.

Furthermore, immunofluorescence co‐localization analysis revealed that, compared with sham controls, KIM‐1 expression in proximal tubular epithelial cells was markedly upregulated in AKI models, and CeAst@MK showed pronounced co‐localization with KIM‐1⁺ tubules (Figure S5, Supporting Information). This indicates that the nanoparticles selectively accumulate in damaged tubular regions, consistent with the function of the KTP on their surface, which specifically recognizes upregulated KIM‐1 in injured kidneys and enhances lesion‐site targeting and uptake. In summary, CeAst@MK demonstrates efficient immune evasion and inflammation‐specific targeting, as well as precise localization to injured renal tissues through macrophage membrane cloaking and KTP functionalization. This dual‐targeting strategy enhances therapeutic accumulation while minimizing off‐target effects, offering a promising platform for precision therapy in AKI.

### 2.5 Cytoprotective Capacity of CeAst@MK

**Figure 4:**
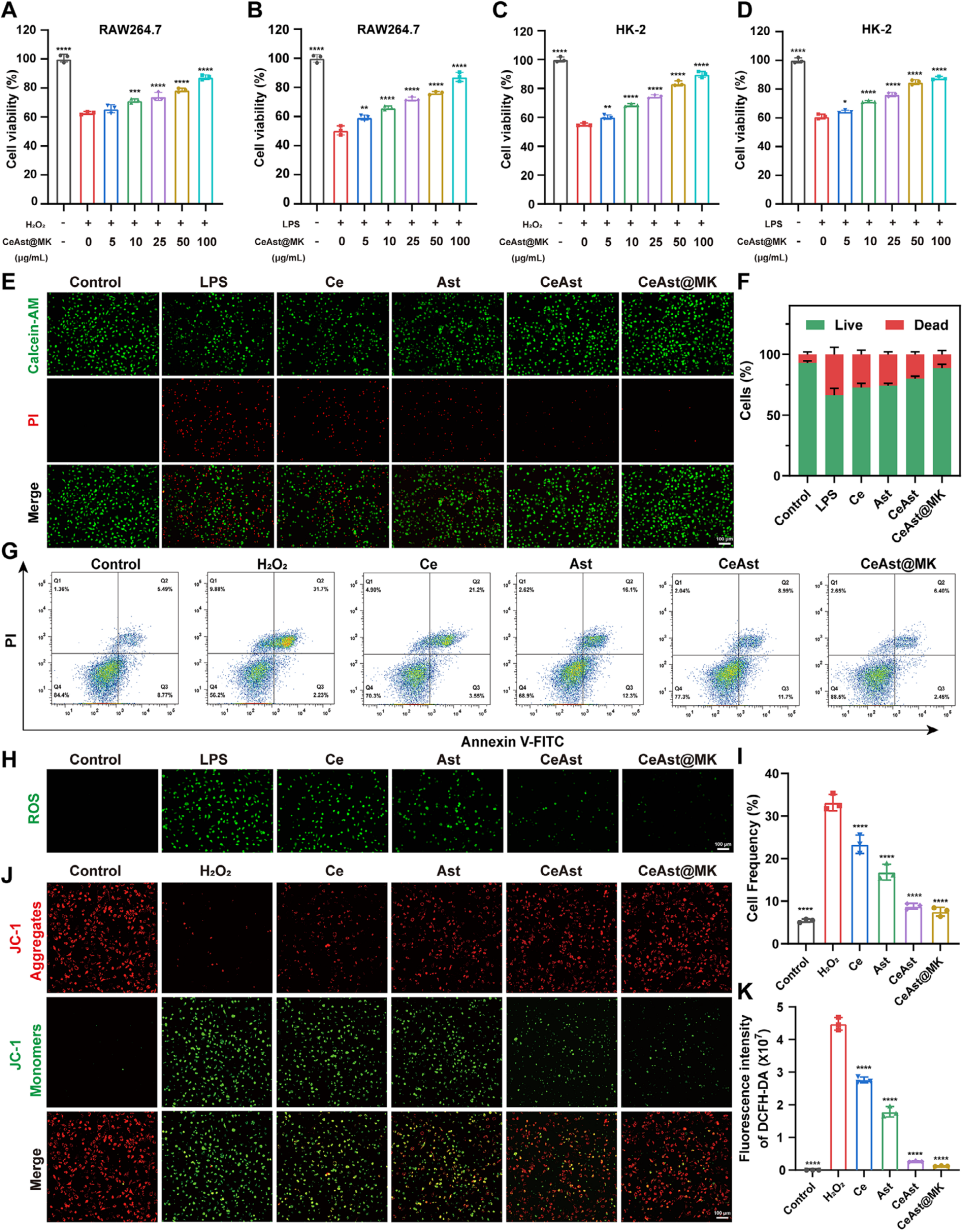
Protective effects of CeAst@MK against cellular oxidative damage. A–D) Viability of RAW264.7 and HK‐2 cells treated with graded concentrations of CeAst@MK followed by LPS or H_2_O_2_ challenge (*n* = 3). E, F) Calcein‐AM/PI staining of HK‐2 cells under different treatments and quantitative analysis (*n* = 3). G,I) Flow cytometric Annexin V/PI analysis and quantification of apoptosis in HK‐2 cells after different treatments (*n* = 3). H,K) DCFH‐DA staining of intracellular ROS in HK‐2 cells under different conditions and corresponding fluorescence quantification (*n* = 3). J) JC‐1 staining images showing mitochondrial membrane potential in HK‐2 cells after treatments (*n* = 3). The data were shown as mean ± SD (*n* = 3). Compared to the model group, *p < 0.05, **p < 0.01, ***p < 0.001, ****p < 0.0001, ns indicates *p* > 0.05, by one‐way ANOVA.

Having established the robust ROS‐scavenging capability of CeAst@MK, we next evaluated its biosafety and protective efficacy in cellular models of oxidative injury. RAW264.7 cells and HK‐2 renal epithelial cells were incubated with increasing concentrations of CeAst@MK (12.5–400 µg mL^−1^) for 24 h. CCK‐8 assays demonstrated negligible cytotoxicity even at the highest tested dose, confirming its excellent biocompatibility and suitability for biomedical applications (Figure S6, Supporting Information). To mimic oxidative stress conditions, cells were challenged with H_2_O_2_ (400 µm) or LPS (1 µg mL^−1^). Pretreatment with 100 µg mL^−1^ CeAst@MK significantly restored cell viability, enhancing RAW264.7 cell survival from 50.5% to 87.3% following LPS stimulation and rescuing HK‐2 cells from 55.1% to 89.8% after H_2_O_2_ exposure (**Figure** **4**A–D), suggesting pronounced cytoprotection against both inflammatory and oxidative insults.

Calcein‐AM/PI viability staining corroborated these findings, demonstrating markedly reduced cell death in CeAst@MK treated HK‐2 cells (Figure 4E,F), with similar protective effects also observed in RAW264.7 cells (Figure S7, Supporting Information). Flow cytometry further confirmed the anti‐apoptotic activity, revealing a significant reduction in both early apoptosis and necrosis in CeAst@MK treated HK‐2 cells (Figure 4G,I). Intracellular ROS levels were assessed using DCFH‐DA probes. Compared with untreated controls, CeAst@MK treatment led to a substantial reduction in ROS accumulation in both HK‐2 and RAW264.7 cells (Figure 4H,K; Figure S8, Supporting Information), outperforming Ce^3^⁺ or Ast alone, highlighting the synergistic antioxidant functionality of the nanoassembly. Given the close link between oxidative stress and mitochondrial dysfunction, we next assessed changes in mitochondrial membrane potential (MMP) using JC‐1 staining.^[^ ^32^ ^]^ H_2_O_2_ challenge induced marked MMP depolarization (loss of red and gain of green fluorescence), indicative of early apoptosis. CeAst@MK treatment effectively reversed this trend, preserving MMP integrity and thereby protecting mitochondrial function (Figure 4J).

**Figure 5:**
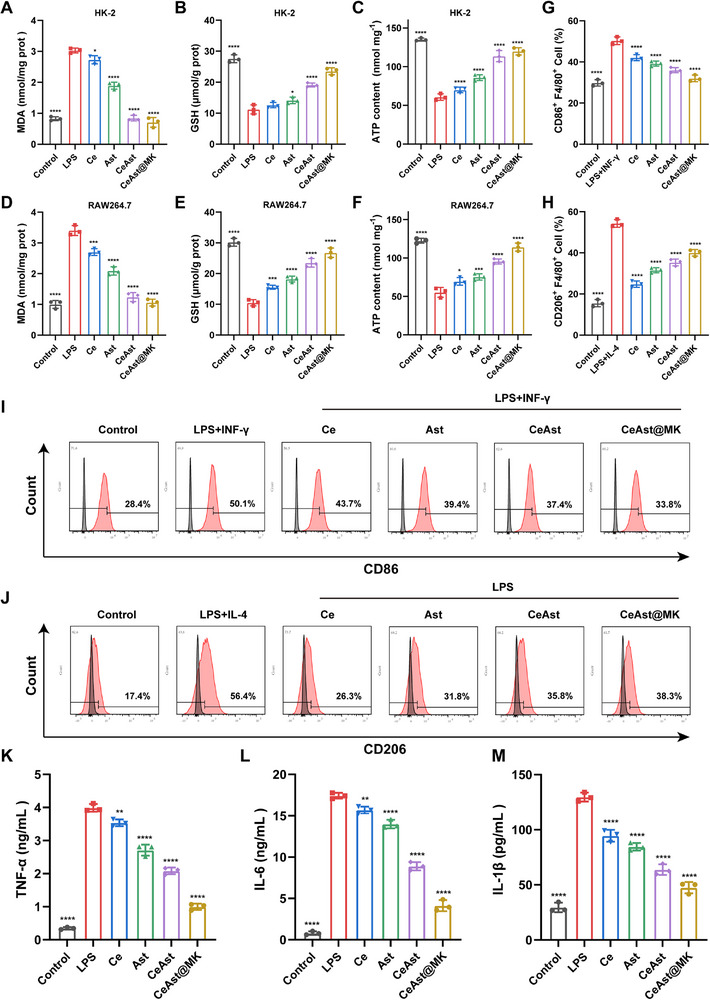
Anti‐inflammatory effects of CeAst@MK in LPS‐stimulated cells. A–C) Quantification of lipid peroxidation, GSH, and ATP levels in HK‐2 cells under various treatments (*n* = 3). D–F) Lipid peroxidation, GSH, and ATP measurements in RAW264.7 cells after different treatments (*n* = 3). G–J) Flow cytometric analysis of CD86 and CD206 expression in RAW264.7 cells and corresponding quantification (*n* = 3). K–M) Secretion of TNF‐α, IL‐6, and IL‐1β in RAW264.7 cells after various treatments (*n* = 3). The data were shown as mean ± SD (*n* = 3). Compared to the model group, *p < 0.05, **p < 0.01, ***p < 0.001, ****p < 0.0001, ns indicates *p* > 0.05, by one‐way ANOVA.

To further explore redox homeostasis, we measured malondialdehyde (MDA) and glutathione (GSH) levels. CeAst@MK significantly suppressed LPS‐induced lipid peroxidation in HK‐2 cells (**Figure** **5**A) and restored intracellular GSH content depleted by oxidative stress (Figure 5B). Moreover, CeAst@MK significantly improved ATP production, which was otherwise impaired under LPS‐induced stress (Figure 5C), underscoring its capacity to mitigate oxidative stress‐related metabolic dysfunction. Parallel effects were observed in RAW264.7 cells (Figures 5D–F). Taken together, CeAst@MK demonstrates potent cytoprotection, efficient intracellular ROS scavenging, and preservation of mitochondrial and metabolic function, supporting its promise as a multifunctional nanotherapeutic for AKI and related oxidative pathologies.

### 2.6 Anti‐Inflammatory Effect of CeAst@MK

Given the critical role of oxidative stress in driving macrophage polarization during AKI, we further investigated the modulatory effects of CeAst@MK on macrophage phenotypes in vitro. Macrophages, as pivotal innate immune cells, orchestrate host defense, immune homeostasis, and tissue repair through remarkable phenotypic plasticity.^[^ ^33^, ^34^ ^]^ They polarize into pro‐inflammatory M1 or anti‐inflammatory M2 states, with M1 macrophages secreting cytokines such as TNF‐α and IL‐6 to amplify inflammation, while M2 macrophages facilitate immunosuppression and tissue regeneration.^[^ ^35^, ^36^ ^]^ Using LPS and IFN‐γ to induce M1 polarization in RAW264.7 cells, flow cytometry revealed that CeAst@MK treatment significantly reduced the proportion of CD86⁺ M1 macrophages, indicating effective suppression of pro‐inflammatory activation (Figure 5G,I).^[^ ^37^ ^]^ Conversely, LPS combined with IL‐4 induced M2 polarization, where CeAst@MK markedly elevated CD206⁺ M2 macrophage populations to 38.3%, outperforming Ce^3^⁺ treatment alone (26.3%) (Figure 5H,J). These data demonstrate CeAst@MK's potent ability to reprogram macrophages toward a reparative phenotype, underscoring the advantage of metal–natural product coordination nanoplatforms in anti‐inflammatory therapy.

Moreover, ELISA assays showed CeAst@MK significantly attenuated LPS‐induced secretion of pro‐inflammatory cytokines TNF‐α, IL‐6, and IL‐1β, surpassing effects observed with equivalent doses of Ce^3+^ or Ast alone (Figure 5K–M). This superior anti‐inflammatory efficacy likely stems from CeAst@MK's synergistic ROS scavenging and macrophage polarization modulation. In conclusion, CeAst@MK robustly inhibits M1 activation, promotes M2 polarization, and suppresses pro‐inflammatory cytokines, highlighting its immunomodulatory and anti‐inflammatory potential.

### 2.7 In Vivo Effects of CeAst@MK on IRI‐AKI Mice

Building on the potent antioxidant and cytoprotective effects of CeAst@MK in vitro, we next assessed its therapeutic potential in an AKI mouse model. AKI is a severe clinical condition with high morbidity and mortality, but limited effective treatments. Oxidative stress is increasingly recognized as a central driver of AKI pathogenesis, highlighting antioxidant intervention as a promising strategy.^[^ ^38^, ^39^ ^]^ IRI, a major cause of AKI, provokes excessive ROS production during early reperfusion due to mitochondrial electron transport chain dysfunction. The resulting oxidative burst induces lipid, protein, and DNA damage, disrupts MMP, and activates apoptotic pathways, collectively leading to tubular epithelial cell death and renal dysfunction.^[^ ^40^ ^]^

**Figure 6:**
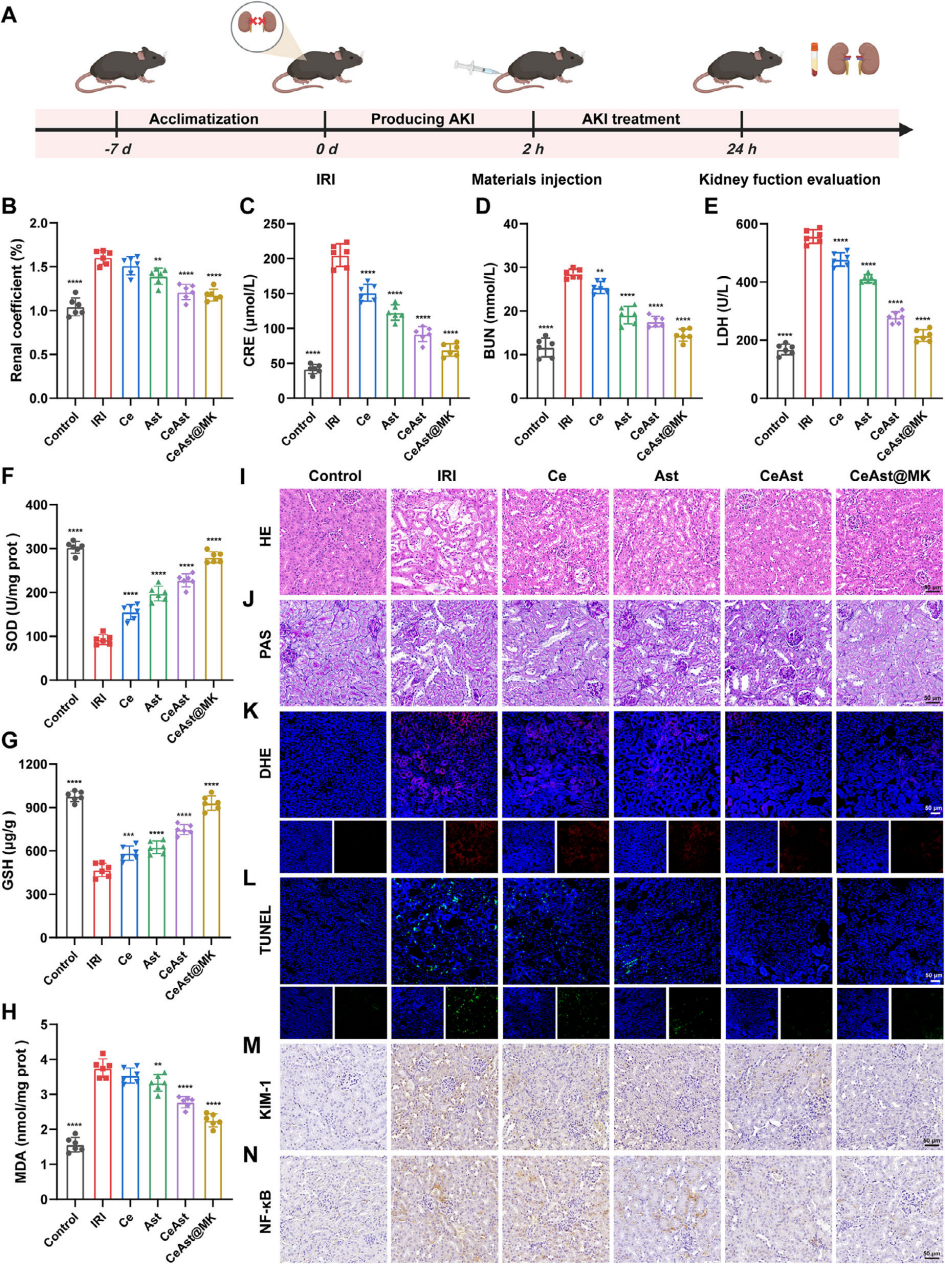
Therapeutic effects of CeAst@MK in IRI‐AKI mice. A) Schematic of IRI‐AKI model establishment and treatment schedule. B) Kidney indices at 24 h. C–E) Serum CRE, BUN, and LDH levels at 24 h (*n* = 6). F–H) Renal SOD, GSH, and MDA levels at 24 h (*n* = 6). I,J) Representative H&E and PAS staining of kidney sections (*n* = 6). K,L) DHE and TUNEL staining of renal tissues at 24 h (*n* = 6). M,N) Representative KIM‐1 and NF‐κB immunohistochemistry in kidneys (*n* = 6). The data were shown as mean ± SD (*n* = 6). Compared to the model group, *p < 0.05, **p < 0.01, ***p < 0.001, ****p < 0.0001, ns indicates *p* > 0.05, by one‐way ANOVA.

To evaluate the therapeutic effects of CeAst@MK in IRI‐induced AKI, we established a bilateral renal pedicle clamping model in mice (**Figure** **6**A). Briefly, both renal arteries were occluded for 25 min using nontraumatic vascular clamps, followed by reperfusion and tail vein administration of the indicated formulations 2 h post‐injury.^[^ ^41^ ^]^ As shown in Figure S9 (Supporting Information), the 7‐day survival rate in the IRI group was only 22.7%, while CeAst@MK treatment significantly improved survival to 83.3%. This was superior to the Ast (45.4%) and CeAst (71.4%) groups, underscoring the synergistic therapeutic potential of CeAst@MK. The kidney index is a commonly used indicator of renal swelling and tissue damage in AKI models.^[^ ^42^ ^]^ The IRI group exhibited substantial renal enlargement and a markedly increased kidney index, reflecting severe tissue injury. In contrast, CeAst@MK treatment significantly reduced kidney index values toward normal levels, outperforming the Ast group (Figure 6B), indicating effective alleviation of edema and tissue damage.

Glomerular filtration was evaluated by measuring serum creatinine (CRE) and blood urea nitrogen (BUN) levels. Both markers were dramatically elevated in IRI mice, confirming severe renal impairment (Figure 6C,D). CeAst@MK significantly lowered BUN and CRE to near normal levels, surpassing the efficacy of Ast alone and further validating its nephroprotective effects. Lactate dehydrogenase (LDH), released into the circulation following membrane disruption, is a sensitive indicator of cellular injury. In AKI, inflammatory responses and immune cell infiltration exacerbate epithelial injury, promoting LDH release.^[^ ^43^ ^]^ As shown in Figure 6E, serum LDH in the IRI group increased to 3.3‐fold of the control, indicating extensive membrane disruption. CeAst@MK markedly reduced LDH leakage, outperforming both the model and Ast groups, and approaching baseline levels, highlighting its strong membrane‐stabilizing capability.

SOD and GSH are key constituents of the intrinsic antioxidant defense network. In AKI, persistent oxidative stress depletes these protective molecules, weakening mitochondrial redox buffering capacity. MDA, a lipid peroxidation byproduct, is widely used as a biomarker of oxidative membrane damage. As shown in Figure 6F,G, SOD activity and GSH levels were markedly reduced in IRI mice, while CeAst@MK treatment restored both indicators to near normal levels, indicating redox homeostasis recovery. Meanwhile, MDA levels in the IRI group increased to 2.3‐fold of control, signifying severe lipid peroxidation. CeAst@MK markedly lowered MDA, suggesting effective protection against membrane oxidative damage (Figure 6H).

During the progression of renal diseases, denatured proteins can deposit in the renal tubular lumen and form characteristic cast structures, which may serve as diagnostic markers for pathological alterations.^[^ ^43^ ^]^ Histological analyses further corroborated CeAst@MK's renoprotective effects. H&E staining revealed severe tubular necrosis and cast formation in the IRI group, while CeAst@MK treated kidneys exhibited preserved architecture with reduced structural disorganization (Figure 6I). Periodic acid Schiff (PAS) staining showed disrupted brush borders and glomerular injury in IRI mice, which were significantly restored by CeAst@MK treatment, with more intact tubular and glomerular morphology (Figure 6J). To assess ROS clearance in vivo, dihydroethidium (DHE) staining was performed on kidney sections. As shown in Figure 6K and Figure S10A (Supporting Information), strong red fluorescence was observed in the IRI group, indicating excessive ROS accumulation. CeAst@MK treatment substantially diminished the fluorescence intensity, outperforming Ast, and confirming potent in vivo antioxidant capacity. Given the central role of apoptosis in AKI progression, TUNEL staining was used to evaluate tubular cell apoptosis.^[^ ^44^ ^]^ Compared with the model and CeAst groups, CeAst@MK markedly decreased TUNEL‐positive tubular cells (Figure 6L; Figure S10B, Supporting Information), demonstrating effective suppression of programmed cell death.

Immunohistochemistry was conducted to evaluate renal injury and associated inflammatory responses. KIM‐1, a transmembrane glycoprotein primarily localized to proximal tubular epithelial cells, is strongly induced following renal damage and serves as a sensitive and specific early biomarker of kidney injury. Relative to the control group, the IRI model exhibited a marked increase in KIM‐1 expression (Figure 6M). Notably, treatment with CeAst@MK markedly attenuated KIM‐1 expression in kidney tissues, suggesting that the nanoplatform effectively alleviated IRI‐induced renal injury. In parallel, expression of the inflammatory pathway marker NF‐κB showed a similar trend, further supporting the anti‐inflammatory efficacy of CeAst@MK (Figure 6N). Collectively, these results demonstrate that CeAst@MK effectively mitigates IRI‐induced AKI through ROS scavenging, mitochondrial protection, and anti‐apoptotic mechanisms, highlighting its translational potential as a novel biomimetic antioxidant platform for AKI therapy.

### 2.8 In Vivo Therapeutic Efficacy of CeAst@MK on LPS‐AKI Mice

**Figure 7:**
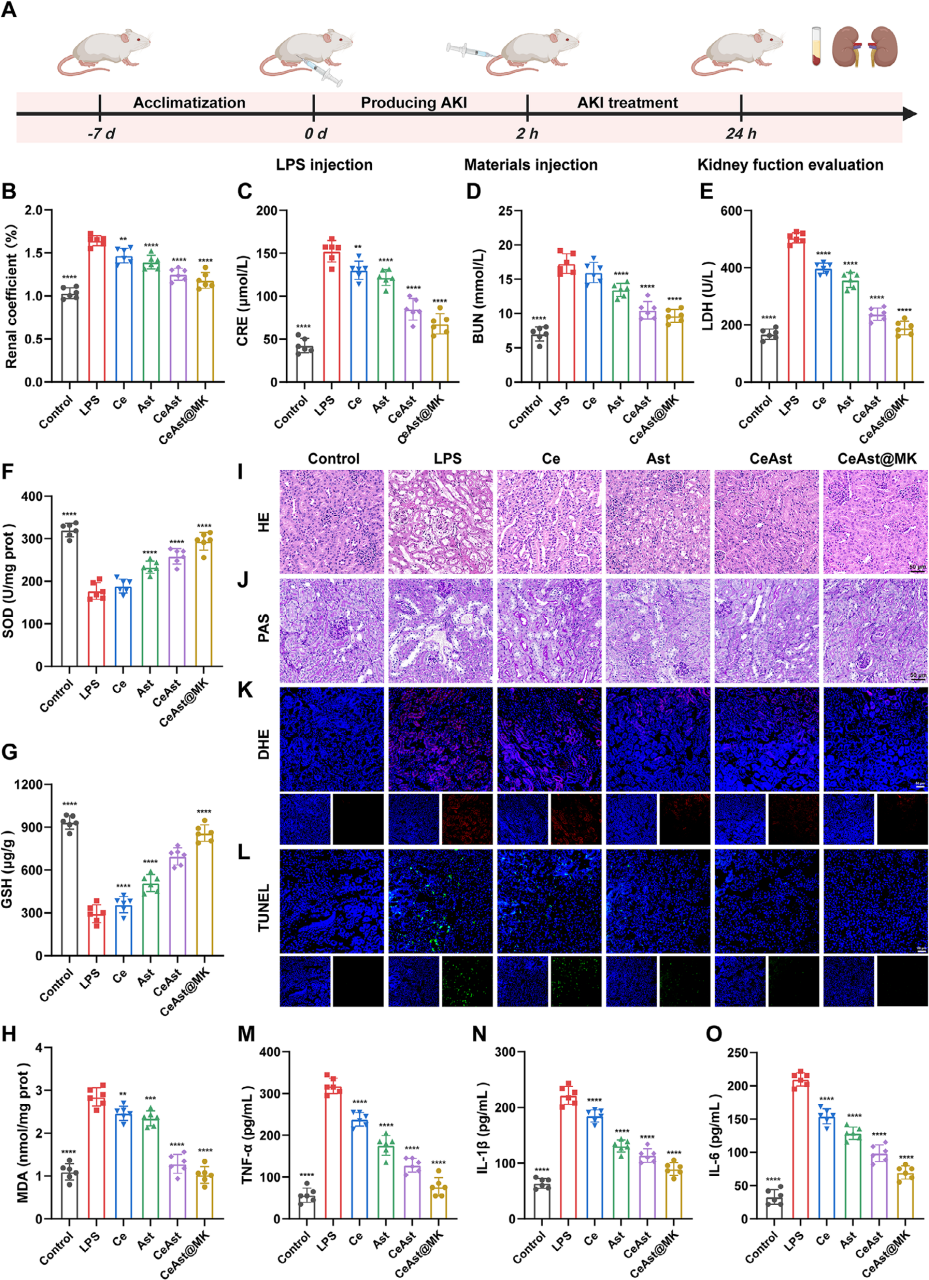
Therapeutic effects of CeAst@MK in LPS‐AKI mice. A) Schematic of LPS‐AKI model establishment and treatment timeline. B) Kidney indices of different treatment groups at 24 h. C–E) Serum CRE, BUN, and LDH levels in experimental groups at 24 h (*n* = 6). F–H) Renal SOD, GSH, and MDA levels at 24 h (*n* = 6). I,J) Representative images H&E and PAS staining of kidney sections (*n* = 6). K,L) DHE and TUNEL staining of renal tissues at 24 h (*n* = 6). M–O) Serum TNF‐α, IL‐1β, and IL‐6 concentrations (*n* = 6). The data were presented as mean ± SD (*n* = 6). Compared to the model group, *p < 0.05, **p < 0.01, ***p < 0.001, ****p < 0.0001, ns indicates *p* > 0.05, by one‐way ANOVA.

As illustrated in **Figure** **7**A, an AKI model was induced in BALB/c mice via intraperitoneal administration of lipopolysaccharide (LPS, 10 mg·kg^−1^), while untreated healthy mice were used as controls. Two hours after LPS administration, various formulations were administered intravenously to initiate treatment. Consistent with its efficacy in the IRI‐induced AKI model, CeAst@MK exhibited potent renoprotective effects in the LPS‐induced model. Treatment significantly reduced kidney index, serum BUN, CRE, and LDH levels, restoring them to near normal values (Figure 7B–E), suggesting effective mitigation of LPS‐induced renal dysfunction. Further mechanistic insights were obtained via oxidative stress biomarkers. Compared to the model and Ast‐treated groups, CeAst@MK significantly increased SOD activity and GSH levels, while reducing MDA, a marker of lipid peroxidation (Figure 7F–H). These results suggest that CeAst@MK enhances endogenous antioxidant defenses while protecting against ROS‐induced membrane damage.

Histopathological analysis supported these functional findings. H&E staining revealed severe tubular disruption and cast formation in the model and Ce‐treated groups, while CeAst@MK markedly preserved tubular architecture and reduced tissue damage (Figure 7I). Similarly, PAS staining confirmed pronounced glomerular and tubular injury in the model group, which was notably reversed in the CeAst@MK‐treated mice, with cellular morphology closely resembling that of healthy controls (Figure 7J). To assess oxidative stress, DHE staining was performed. LPS challenge caused robust red fluorescence accumulation in renal tissue, indicating a high ROS burden. CeAst@MK significantly suppressed this signal, demonstrating superior in vivo ROS scavenging ability (Figure 7K; Figure S11A, Supporting Information). TUNEL staining revealed widespread apoptosis in LPS‐challenged kidneys, as evidenced by strong green fluorescence, which was substantially attenuated following CeAst@MK administration, demonstrating its antiapoptotic activity (Figure 7L; Figure S11B, Supporting Information). Consistently, immunohistochemistry showed pronounced upregulation of KIM‐1 in the IRI model, whereas CeAst@MK treatment markedly reduced its expression, reflecting a protective effect against renal injury (Figure S12, Supporting Information). Similarly, NF‐κB expression exhibited a comparable trend, supporting the anti‐inflammatory activity of CeAst@MK.

During AKI, renal tubular epithelial cells release large amounts of chemokines that attract immune cells, particularly M1 macrophages, thereby amplifying tissue injury through the secretion of pro‐inflammatory cytokines. Consistent with this, ELISA measurements showed that serum TNF‐α, IL‐1β, and IL‐6 levels were markedly elevated in LPS‐induced AKI mice, whereas CeAst@MK treatment significantly suppressed their expression (Figure 7M–O). Similarly, qRT‐PCR analysis demonstrated that LPS markedly upregulated the mRNA levels of these proinflammatory cytokines in renal tissues. Notably, CeAst@MK treatment effectively suppressed the overexpression of these inflammatory mediators (Figure S13, Supporting Information). Collectively, these findings demonstrate that CeAst@MK effectively alleviates LPS‐induced AKI through combined mechanisms involving oxidative stress suppression, apoptosis inhibition, and restoration of renal function, highlighting its promise as a clinically translatable nanotherapeutic for AKI intervention.

### 2.9 Macrophage Phenotypic Changes in the Kidney

**Figure 8:**
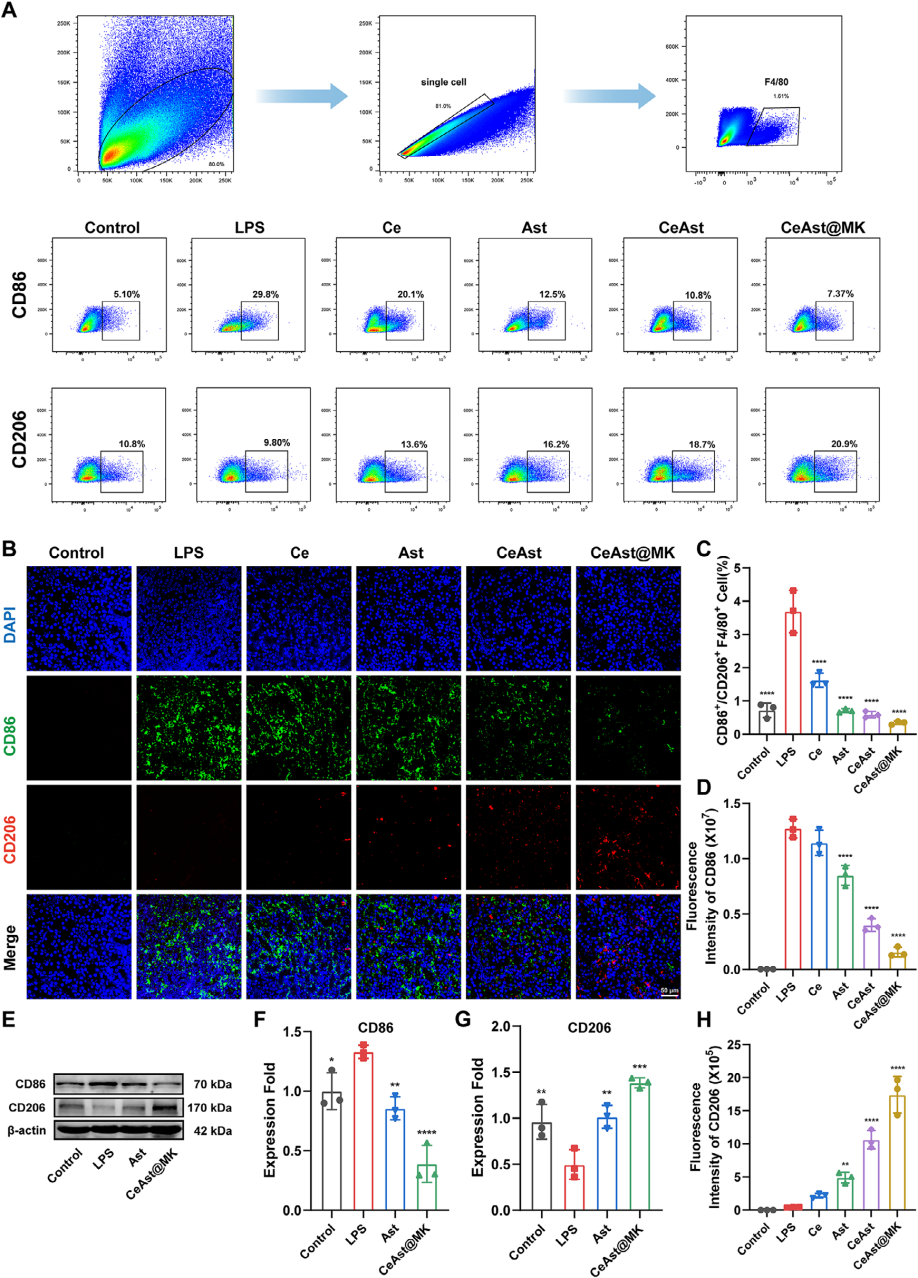
Macrophage polarization in the kidney. A) Flow cytometric analysis of CD86 and CD206 in renal macrophages. B) Representative immunofluorescence images of CD86 and CD206 in kidney sections. C) Quantification of the CD86/CD206 ratio from flow cytometry (*n* = 3). D,H) Fluorescence intensity of CD86 and CD206 in renal tissues (*n* = 3). E–G) Western blot analysis of CD86 and CD206 protein levels with semi‐quantitative densitometry (*n* = 3). The data were presented as mean ± SD (*n* = 3). Compared to the model group, *p < 0.05, **p < 0.01, ***p < 0.001, ****p < 0.0001, ns indicates *p* > 0.05, by one‐way ANOVA.

Macrophages serve as key innate immune sentinels, orchestrating pathogen clearance, phagocytosis, and inflammatory regulation. Their functional states are shaped by environmental cues. M1 macrophages exhibit a pro‐inflammatory phenotype marked by TNF‐α, IL‐1β, and IL‐6 secretion, whereas M2 macrophages produce IL‐10 and facilitate tissue repair.^[^ ^45^ ^]^ To assess the impact of CeAst@MK on macrophage polarization in vivo, renal immune cells were analyzed by flow cytometry. LPS challenge markedly increased the CD86⁺/CD206⁺ ratio, indicating enhanced M1 polarization, while CeAst and CeAst@MK treatment significantly reduced this shift (**Figure** **8**A,C). Immunofluorescence confirmed reduced CD86 and elevated CD206 expression in CeAst@MK‐treated kidneys (Figure 8B,D,H). Consistently, Western blot analysis demonstrated downregulation of CD86 and upregulation of CD206 following treatment (Figure 8E–G).

Flow cytometry analysis revealed the dynamic changes in renal macrophage phenotypes during AKI (Figures S14 and S15, Supporting Information). In both models, compared with the control group, the proportion of CD86⁺ M1 macrophages significantly increased at 12–24 h after injury, whereas CD206⁺ M2 macrophages were markedly elevated at 48–72 h. Treatment with CeAst@MK reduced the proportion of CD86⁺ macrophages while progressively increasing the proportion of CD206⁺ macrophages over time. These results are consistent with previous reports showing that early‐stage AKI is dominated by M1‐driven pro‐inflammatory responses, which subsequently shift toward an M2 phenotype associated with tissue repair.^[^ ^46^, ^47^ ^]^ Collectively, these findings demonstrate that CeAst@MK effectively suppresses excessive early inflammation and promotes macrophage polarization toward a reparative M2 phenotype, thereby orchestrating the renal immune microenvironment.

### 2.10 Cellular Transcriptomic of CeAst@MK on LPS‐AKI Mice

**Figure 9:**
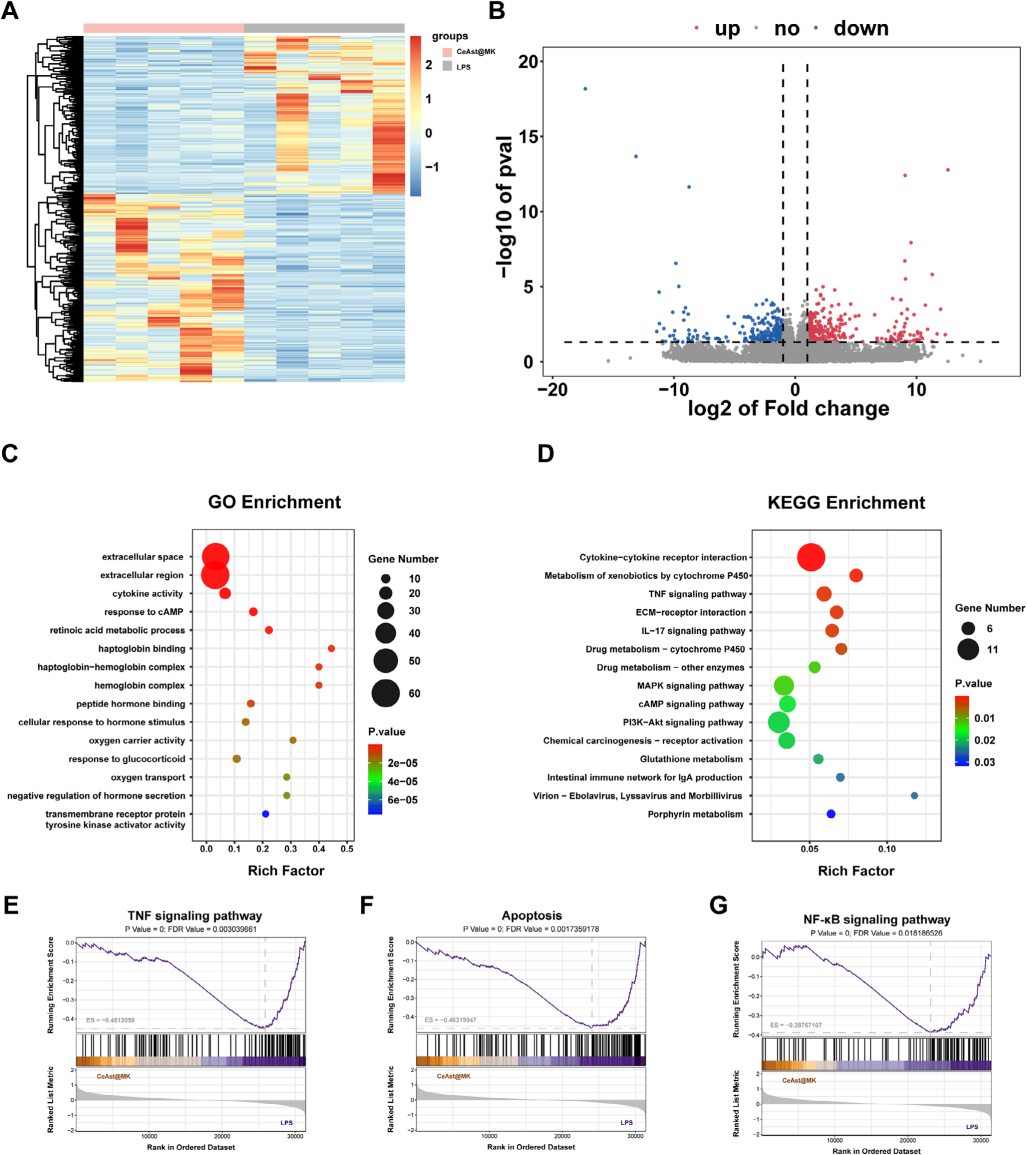
Transcriptomic analysis of the renal effects of CeAst@MK in LPS‐AKI mice. A) Heatmap of DEGs between the LPS groups and CeAst@MK treated groups (*n* = 5). B) Volcano plot of DEGs between the LPS groups and CeAst@MK treated groups. C) GO enrichment analysis of the LPS groups and CeAst@MK treated groups. D) KEGG pathway enrichment analysis of the LPS groups and CeAst@MK treated groups. E–G) GSEA enrichment analysis of the LPS groups and CeAst@MK treated groups.

To further elucidate the molecular mechanisms by which CeAst@MK mitigates AKI, RNA sequencing (RNA‐seq) was performed on renal tissues to characterize the global transcriptional alterations induced by treatment. Heatmap analysis revealed pronounced transcriptomic alterations between the LPS model and CeAst@MK‐treated groups (**Figure** **9**A). In total, 572 differentially expressed genes (DEGs) were identified, including 311 upregulated and 261 downregulated genes in the CeAst@MK group relative to the model group (Figure 9B). These DEGs were functionally associated with mitochondrial dynamics, inflammatory signaling, apoptosis, and metabolic regulation (Figure S16, Supporting Information). GO enrichment analysis demonstrated significant enrichment of DEGs in the extracellular region, cytokine activity, hormone response, oxygen transport, and retinoic acid metabolism, collectively indicating that CeAst@MK mediates its therapeutic efficacy through suppressing inflammatory signaling, improving mitochondrial and oxygen metabolism, and modulating hormone‐related pathways (Figure 9C). Together, these findings indicate that CeAst@MK alleviates LPS‐induced AKI by suppressing inflammatory signaling, improving mitochondrial and oxygen metabolism, modulating hormone‐related pathways, and promoting tissue repair, thereby restoring immune homeostasis and reducing renal injury. To further investigate the signaling pathways modulated by CeAst@MK, Kyoto Encyclopedia of Genes and Genomes (KEGG) analysis was conducted on DEGs between the LPS and CeAst@MK groups (Figure 9D). Pathways related to cytokine–cytokine receptor interaction, TNF signaling, IL‐17 signaling, and chemokine‐mediated inflammation were significantly downregulated, indicating attenuation of pro‐inflammatory signaling. In parallel, pathways associated with ECM–receptor interactions, PI3K/Akt, and MAPK signaling, key regulators of cell survival, oxidative stress response, and tissue regeneration, were enriched. These results suggest that CeAst@MK confers renoprotection through integrated modulation of immune and metabolic pathways, targeting critical mechanisms underlying AKI progression and recovery.

Gene set enrichment analysis (GSEA) further confirmed the multi‐faceted regulatory effects of CeAst@MK (Figure 9E–G). Notably, canonical pro‐inflammatory and pro‐apoptotic pathways, including TNF, apoptosis, and the NF‐κB signaling pathway, were markedly inhibited, indicating restoration of mitochondrial function and cellular energy metabolism in renal epithelial cells. Collectively, these transcriptional reprogramming events collectively contribute to inflammation attenuation and tissue regeneration. PPI network analysis revealed the top 10 hub genes with the highest centrality, predominantly associated with the regulation of inflammatory cytokines, immune cell activation, and leukocyte adhesion and migration (Figure S17, Supporting Information). This network analysis highlights the multi‐target immunomodulatory potential of CeAst@MK in restoring renal immune homeostasis. Collectively, these results demonstrate that CeAst@MK mitigates AKI through coordinated suppression of pro‐inflammatory signaling, reprogramming of immune responses, and reinforcement of mitochondrial function, offering a robust and multifaceted therapeutic strategy for AKI.

### 2.11 Therapeutic Mechanism of CeAst@MK on LPS‐AKI Mice

The kidney, as a highly metabolically active organ, relies strongly on adequate energy supply and precise regulation of cellular homeostasis. AKI represents a clinical condition marked by a sudden decline in renal function, typically associated with elevated oxidative stress, excessive inflammation, and extensive apoptosis of tubular epithelial cells. Among the signaling cascades implicated in AKI progression, the PI3K/Akt pathway and the mitochondria‐mediated intrinsic apoptotic cascade serve as pivotal regulators of cell survival and death.^[^ ^48^ ^]^ The PI3K/Akt signaling pathway is a highly conserved pro‐survival cascade that governs key biological processes, including cell proliferation, metabolism, antioxidative defense, and anti‐apoptotic signaling. Specifically, class I PI3K‐mediated PIP3 generation facilitates Akt membrane translocation and activation, initiating downstream phosphorylation cascades that suppress pro‐apoptotic effectors while enhancing anti‐apoptotic factors. Paradoxically, the AKI microenvironment, characterized by oxidative and inflammatory stress, frequently impairs this protective signaling through Akt phosphorylation suppression, thereby compromising renal cellular repair mechanisms and promoting apoptotic cell death.^[^ ^49^, ^50^ ^]^

In response to cellular insults such as excessive ROS, mitochondrial DNA injury, and calcium imbalance, the mitochondrial apoptotic pathway is triggered. This process is governed by the Bcl‐2 protein family. Under stress conditions, pro‐apoptotic members, including Bax and Bak, undergo conformational activation, oligomerize, and permeabilize the mitochondrial outer membrane, facilitating cytochrome c (Cyt‐c) release into the cytosol. Cyt‐c then initiates apoptosome assembly and sequential caspase activation, culminating in programmed cell death.^[^ ^51^ ^]^ Conversely, anti‐apoptotic proteins like Bcl‐2 counteract this process by restraining Bax activation, with the Bcl‐2/Bax ratio serving as a key determinant of cellular resistance to apoptosis. In AKI, this balance shifts toward Bax, driving mitochondrial dysfunction and tubular epithelial cell injury. Therefore, restoring the Bcl‐2/Bax equilibrium and maintaining mitochondrial membrane integrity are critical therapeutic strategies. In addition, PI3K/Akt signaling intersects with the NF‐κB pathway, where Akt activation suppresses IκB kinase (IKK), thereby preventing NF‐κB nuclear entry and attenuating the transcription of pro‐inflammatory mediators such as TNF‐α and IL‐1β.^[^ ^52^ ^]^ Thus, PI3K/Akt activation exerts dual protective effects by limiting apoptosis and dampening inflammation. To dissect the mechanisms underlying the renoprotective effects of CeAst@MK, we examined molecular endpoints in renal tissues, including PI3K/Akt phosphorylation, Bcl‐2 family and caspase expression, pro‐inflammatory cytokine release, and renal injury markers.

**Figure 10:**
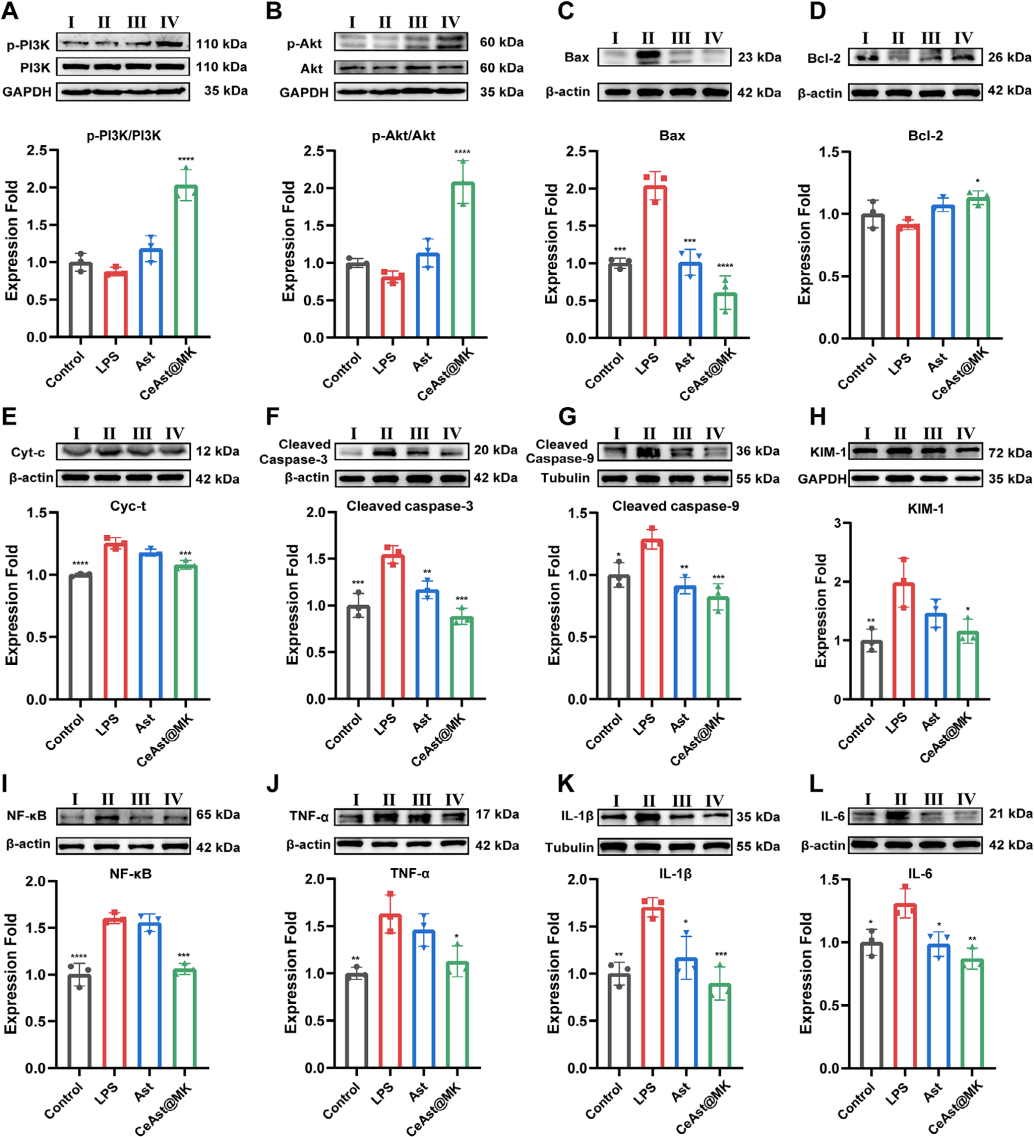
Therapeutic mechanism of CeAst@MK on LPS‐AKI mice. The representative Western blot analysis and semiquantitative analyses of A) PI3K/p‐PI3K, B) Akt/p‐Akt, C) Bax, D) Bcl‐2, E) Cyt‐c, F) Cleaved Caspase‐3, G) Cleaved Caspase‐9, H) KIM‐1, I) NF‐κB, J) TNF‐α, K) IL‐1β, and L) IL‐6 (*n* = 3). I–IV represent the Control group, the LPS group, the Ast group, and the CeAst@MK group, respectively. The data were shown as mean ± SD (*n* = 3). Compared to the model group, *p < 0.05, **p < 0.01, ***p < 0.001, ****p < 0.0001, ns indicates *p* > 0.05, by one‐way ANOVA.

In the LPS‐induced AKI model, a pronounced reduction in phosphorylated PI3K and Akt was detected, reflecting suppression of pro‐survival signaling in renal cells. Treatment with CeAst@MK markedly increased phosphorylation of both PI3K and Akt (**Figure** **10**A,B), indicating reactivation of this protective pathway and enhanced resistance of tubular epithelial cells to injury. Mechanistically, CeAst@MK exerts SOD and CAT‐mimetic activities, efficiently scavenging LPS‐induced ROS and thereby mitigating oxidative damage to membranes and signaling components. The reduction in ROS stabilizes membrane lipids and promotes PIP3 formation, which in turn facilitates PI3K activation and downstream Akt phosphorylation. This signaling cascade suppresses apoptosis and supports epithelial regeneration, highlighting PI3K/Akt activation as a central mechanism of CeAst@MK mediated renoprotection. Excessive mitochondrial ROS further triggered the intrinsic apoptotic pathway. Western blotting revealed elevated Bax and reduced Bcl‐2 expression in the LPS group, consistent with apoptosis activation. CeAst@MK treatment decreased Bax and increased Bcl‐2 (Figure 10C,D), with Ast alone also showing a partial effect. Consequently, the Bax/Bcl‐2 ratio was significantly lowered, indicating effective inhibition of mitochondrial apoptosis. Bax translocation to the mitochondrial outer membrane promotes Cyt‐c release, whereas Bcl‐2 prevents this by restraining Bax activation or sequestering BH3‐only proteins. In the cytosol, Cyt‐c associates with Apaf‐1 to assemble the apoptosome, initiating caspase‐9 activation and subsequent caspase‐3 cleavage. Consistent with Bax/Bcl‐2 modulation, CeAst@MK reduced Cyt‐c release compared to the model group (Figure 10E), further confirming its anti‐apoptotic effect.

We next examined the downstream caspase cascade. Both cleaved caspase‐3 and cleaved caspase‐9 were markedly upregulated in the LPS group, whereas CeAst@MK significantly suppressed their activation, showing stronger inhibition than Ast alone (Figure 10F,G). These results confirm that CeAst@MK blocks mitochondrial apoptosis by regulating Bcl‐2 family proteins and preventing caspase cascade initiation. Inflammation also plays a pivotal role in AKI. CeAst@MK treatment markedly reduced NF‐κB phosphorylation (Figure 10I) and downregulated TNF‐α, IL‐1β, and IL‐6 expression (Figure 10J–L), consistent with its ROS‐scavenging and immunomodulatory functions. In addition, KIM‐1, an early and sensitive marker of tubular damage linked to CKD progression, was significantly elevated after LPS challenge but strongly reduced by CeAst@MK (Figure 10H).^[^ ^53^, ^54^ ^]^ Similarly, neutrophil gelatinase‐associated lipocalin (NGAL), another established biomarker of AKI severity, was upregulated in LPS‐treated mice and reversed by both Ast and CeAst@MK.^[^ ^55^ ^]^ Notably, a single CeAst@MK administration restored NGAL levels close to baseline (Figure S18, Supporting Information), indicating rapid therapeutic efficacy.

In conclusion, CeAst@MK confers robust renoprotection against LPS‐induced AKI through a multi‐pronged mechanism. It scavenges ROS, stabilizes mitochondrial function, inhibits Bcl‐2/Bax‐driven apoptosis and caspase activation, and suppresses NF‐κB‐mediated inflammation. Concurrent downregulation of KIM‐1 and NGAL further highlights its ability to mitigate tubular injury. Collectively, these findings establish CeAst@MK as a promising smart nanotherapeutic that alleviates AKI by orchestrating the PI3K/Akt–mitochondrial apoptosis–NF‐κB axis.

### 2.12 Biosafety Evaluation

Nanomedicine holds great promise for drug delivery; however, concerns over potential nanotoxicity remain a significant barrier to clinical translation. Given the kidney's pivotal role in systemic homeostasis and metabolic waste clearance, evaluating the biocompatibility of nanotherapeutics is particularly critical for their application in AKI. Therefore, we systematically assessed the in vivo safety profile of CeAst@MK. Hemolysis assays demonstrated negligible hemolytic activity across a wide concentration range (25–400 µg·mL^−1^), with preserved red blood cell morphology, confirming excellent hemocompatibility (Figure S19, Supporting Information). Furthermore, serum biochemical analyses revealed that key indicators of renal and hepatic function, including BUN, CRE, alanine aminotransferase (ALT), and aspartate aminotransferase (AST), remained within normal ranges and showed no significant differences compared to PBS‐treated controls following 7 days of intravenous injection with CeAst or CeAst@MK (Figure S20, Supporting Information). Consistently, routine hematological parameters also remained within physiological limits across all groups, further supporting systemic safety (Figure S21, Supporting Information). Histopathological evaluation of major organs (heart, liver, spleen, lung, and kidney) via H&E staining revealed no observable tissue damage or inflammatory infiltration following CeAst@MK treatment (Figure S22, Supporting Information). Collectively, these findings confirm that CeAst@MK exhibits excellent biocompatibility and in vivo safety, supporting its potential for clinical translation in the treatment of AKI and other inflammation‐related kidney disorders.

## 3 Conclusion

In conclusion, we have developed a multifunctional bioinspired nanotherapeutic platform, CeAst@MK, integrating antioxidant, anti‐inflammatory, and targeted delivery functionalities for precision intervention in AKI. The core coordination complex, CeAst, formed by Ce^3^⁺ and the natural flavonoid Ast, enables potent ROS scavenging and immunomodulation. Further cloaking with macrophage cell membranes and surface modification with a KTP confer immune evasion, inflammation‐specific accumulation, and lesion‐directed delivery. CeAst@MK exhibits pH‐responsive release behavior, allowing rapid therapeutic activation within the mildly acidic microenvironment of injured renal tissues. Both in vitro and in vivo experiments demonstrate that CeAst@MK significantly improves renal function, alleviates oxidative stress and inflammatory responses, promotes macrophage polarization toward the M2 phenotype, and orchestrates multi‐pathway regulation via PI3K/Akt and NF‐κB signaling. Moreover, the nanoplatform exhibits excellent biocompatibility and systemic safety. Collectively, CeAst@MK synergistically integrates natural pharmacophores, redox‐active cerium, and biomimetic targeting into a smart, stimuli‐responsive nanoplatform with multi‐targeted intervention capacity, offering a promising and translatable strategy for the precise treatment of AKI and other oxidative stress‐associated inflammatory disorders.

## 4 Experimental Section

### Materials

Ce(NO_3_)_3_·6H_2_O and PVP (Mw = 40 000 Da) were obtained from Aladdin Biochemical Technology Co., Ltd. (Shanghai, China). Astragalin (Ast) and IL‐4 were purchased from MedChemExpress (MCE), and LPS from Sigma–Aldrich (USA). DPPH^●^ and ABTS⁺· were obtained from Macklin (Shanghai, China). KTP (Lys‐Cys‐Ser‐Ala‐Val‐Pro‐Leu‐Cys; H_2_N‐KCSAVPLC; purity ≥95%) was synthesized by China Peptides Co., Ltd. (Shanghai, China). Calcein/PI, Annexin V‐FITC, DHE, ATP, and Dil kits were obtained from Beyotime Biotechnology (Shanghai, China). JC‐1, MDA, and LDH kits from Solarbio (Beijing, China). Commercial kits for superoxide anion, SOD, CAT, ROS, GSH, BUN, CRE, AST, and ALT assays were from Nanjing Jiancheng Bioengineering Institute. APC‐anti‐CD206, PE‐anti‐CD86, FITC‐anti‐F4/80 antibodies, and TUNEL kits were obtained from Elabscience (Wuhan, China). ELISA kits were purchased from Thermo Fisher Scientific. Primary antibodies against P‐PI3K, PI3K, P‐Akt, Akt, Bax, Bcl‐2, Caspase‐3, Caspase‐9, Cyt‐c, TNF‐α, IL‐6, IL‐1β, KIM‐1, integrin β1, integrin α4, CD47, β‐actin, and GAPDH were supplied by Proteintech (Wuhan, China).

### Synthesis of CeAst

CeAst nanoparticles were synthesized via a self‐assembly approach. Briefly, Ce(NO_3_)_3_·6H_2_O (27.45 mg mL^−1^, ethanol) was added dropwise into a PVP solution (20 mg mL^−1^, ethanol) under vigorous stirring, followed by the introduction of Ast (5 mg mL^−1^) to initiate assembly. The reaction proceeded for 3 h at room temperature with continuous agitation. Unreacted materials were removed by centrifugation (8000 g, 10 min), and the nanoparticles were purified by dialysis against deionized water (MWCO 44 kDa) for 48 h. The resulting CeAst suspension was stored at 4 °C for subsequent characterization and experiments.

### Isolation of Engineered Macrophage Cell Membranes

M2 polarization of RAW 264.7 cells was induced by treatment with recombinant IL‐4 (20 ng mL^−1^) for 24 h. Membranes were subsequently isolated using a standard extraction protocol.^[^ ^56^ ^]^ Cells were collected, centrifuged at 600 g for 5 min at 4 °C to remove medium, washed with PBS, and pelleted. The pellet was resuspended in extraction buffer A containing 1 mm PMSF, incubated on ice for 15 min, and subjected to three freeze–thaw cycles for disruption. Lysates were centrifuged at 700 g for 10 min at 4 °C to remove nuclei and debris, followed by ultracentrifugation at 14 000 g for 30 min to obtain the membrane fraction. Isolated membranes were stored at −80 °C. Protein concentration was quantified by BCA assay, and membrane protein composition was assessed by SDS‐PAGE with Coomassie brilliant blue staining and imaged using a Bio‐Rad gel documentation system.

### Preparation of CeAst@MK

To construct macrophage membrane‐coated CeAst nanoparticles (CeAst@MK), macrophage‐derived vesicles were first obtained by extruding a 1 mg mL^−1^ membrane suspension successively through 400 nm and 200 nm polycarbonate membranes with a mini‐extruder, yielding uniform vesicles. CeAst nanoparticles (1 mg mL^−1^) were then blended with the vesicles (1 mg mL^−1^) at a 1:1 volume ratio and sonicated in an ice bath for 10 min. The resulting mixture was subsequently passed 30 times through a 200 nm membrane to generate CeAst@M nanoparticles, which were isolated by centrifugation at 12 000 g for 10 min. The collected CeAst@M nanoparticles (1 mg mL^−1^) were further incubated with KTP (0.2 mg mL^−1^) under sonication for 1 h to promote peptide insertion. CeAst@MK nanoparticles were then recovered by centrifugation (12 000 g, 10 min, 4 °C). The incorporation efficiency of KTP was determined by high‐performance liquid chromatography (HPLC) by quantifying the residual free peptides in the supernatant.

### Characterization

The physicochemical characteristics of CeAst@MK were comprehensively characterized. Morphology was examined by TEM (Hitachi HT7700, Japan), elemental composition and oxidation states by XPS (Thermo Fisher ESCALAB 250Xi, USA), and crystallinity by XRD (Bruker D8 Advance, Germany). Fluorescence signals were visualized using CLSM (Olympus FluoView 1200, Japan), with cellular analysis performed by flow cytometry (BD FACSVerse, USA). Molecular bonding was further confirmed by FT‐IR (Shimadzu FTIR‐8300, Japan).

### Radical Scavenging Activity of CeAst@MK

O_2_^•^ **^−^** scavenging activity was assessed using a commercial SOD assay kit. Various concentrations of CeAst@MK were mixed with the reaction system in a 96‐well plate, followed by incubation at 37 °C for 30 min and absorbance measurement at 450 nm.

•OH scavenging capacity was determined with a hydroxyl radical detection kit. After mixing different concentrations of CeAst@MK with reaction reagents, the mixture was incubated for 20 min before measuring absorbance at 550 nm.

H_2_O_2_ scavenging efficiency was evaluated using a catalase activity assay kit. Samples were incubated with H_2_O_2_ at 37 °C for 24 h, with scavenging efficiency calculated based on absorbance at 405 nm.

ABTS^•^ **^+^** scavenging activity was tested by first generating radicals through 12‐h dark incubation of 7 mm ABTS with 2.45 mm potassium persulfate. After adding CeAst@MK samples, the mixture was incubated in the dark for 20 min before measuring absorbance at 734 nm.

DPPH^●^ scavenging activity was examined by mixing equal volumes of 125 µm DPPH solution with CeAst@MK, incubating at 37 °C for 30 min, and measuring absorbance at 517 nm.

### Cell Culture

HK‐2 cells were maintained in DMEM/F12 medium containing 10% FBS and 1% penicillin/streptomycin. RAW264.7 cells were cultured in high‐glucose DMEM (Gibco) supplemented with identical additives. All cell lines were incubated at 37 °C in a 5% CO_2_ humidified atmosphere.

### Cellular Uptake

For nanoparticle internalization assessment, RAW264.7 cells were seeded in 12‐well culture plates (5 × 10⁴ cells per well) and cultured overnight. Cells were then incubated with DiI‐labeled CeAst or CeAst@MK nanoparticles for 6 h at 37 °C, followed by three PBS washes, fixation with 4% paraformaldehyde for 10 min, and DAPI nuclear staining before CLSM imaging. For inflammatory condition studies, HK‐2 cells were pretreated with 1 µg mL^−1^ LPS for 1 h prior to 6‐h incubation with nanoparticles at 37 °C. After performing the same washing and staining protocol, cellular internalization was evaluated by CLSM.

### Cell Viability and Cytoprotective Efficacy Assays

RAW264.7 and HK‐2 cells were seeded in 96‐well plates (1 × 10⁴ cells per well) and cultured overnight. To evaluate cytotoxicity, cells were treated with CeAst@MK (12.5–400 µg mL^−1^) for 24 h, followed by CCK‐8 incubation (10%, 2 h) and absorbance measurement at 450 nm. For cytoprotection studies, cells were pretreated with CeAst@MK (12.5–400 µg mL^−1^) for 1 h prior to oxidative stress induction with either 400 µm H_2_O_2_ or 1 µg mL^−1^ LPS, with viability assessed after 24 h using the same CCK‐8 protocol.

### Intracellular ROS Detection

Intracellular ROS was detected using DCFH‐DA. Cells (1 × 10⁶ cells per well) were pretreated with Ce^3^⁺, Ast, CeAst, or CeAst@MK for 1 h before LPS stimulation (1 µg mL^−1^) for 24 h. After DCFH‐DA staining at 37 °C for 30 min and PBS washing, fluorescence was quantified by microscopy.

### Live/Dead Cell Assay

RAW264.7 and HK‐2 cells (1 × 10⁶ cells per well) were pretreated with test compounds Ce^3^⁺ (8 µg mL^−1^), Ast (20 µg mL^−1^), CeAst (80 µg mL^−1^), or CeAst@MK (100 µg mL^−1^) for 1 h prior to oxidative stress induction with 400 µm H_2_O_2_ for 24 h. Cell viability was determined using calcein‐AM/PI double staining at 37 °C for 30 min, followed by fluorescence microscopy.

### Mitochondrial Membrane Potential Assay

Following identical pretreatment conditions for 1 h, cells were challenged with 1 µg mL^−1^ LPS for 24 h to induce mitochondrial impairment. JC‐1 staining was performed at 37 °C for 20 min to visualize membrane potential (ΔΨm) changes via fluorescence microscopy.

### In Vitro Macrophage Polarization Assay

RAW264.7 cells (1 × 10⁶ cells per well) were treated for 24 h with either polarization controls (LPS/IFN‐γ for M1 phenotype or IL‐4 for M2 phenotype) or test materials (Ce^3^⁺, Ast, CeAst, or CeAst@MK), followed by staining with APC‐anti‐CD206, PE‐anti‐CD86, and FITC‐anti‐F4/80 antibodies at 4 °C for 30 min and subsequent flow cytometric analysis of surface marker expression.

### Cytokine Quantification

RAW264.7 cells were pretreated with Ce^3^⁺, Ast, CeAst, or CeAst@MK for 1 h before LPS stimulation (1 µg mL^−1^, 24 h). Culture supernatants were analyzed for TNF‐α, IL‐6, and IL‐1β levels using commercial ELISA kits.

### Hemolysis Assay

Mouse red blood cells were collected, washed, and adjusted to a 4% suspension. Equal volumes (500 µL) of RBC suspension and CeAst@MK at various concentrations (12.5–400 µg mL^−1^) were mixed and incubated at 37 °C for 1 h. Supernatants were collected post‐centrifugation, and absorbance was measured at 542 nm. Hemolysis (%) was calculated accordingly.

### In Vivo Biodistribution

A bilateral renal IRI model was established. Cy5.5‐labeled CeAst@MK was intravenously injected via the tail vein. Sham‐operated mice received the same dose as controls. At 1, 3, 6, 12, and 24 h post‐injection, major organs were harvested and imaged using an IVIS Spectrum (Ex 675 nm / Em 720 nm) to assess biodistribution.

### In Vivo Therapeutic Evaluation in AKI Models

Male BALB/c or C57BL/6 mice (6–8 weeks) were used to establish LPS‐induced (10 mg kg^−1^) or renal IRI (25 min bilateral pedicle clamping) AKI models. All animal experiments were strictly conducted in accordance with national guidelines and regulations for laboratory animals, and were approved by the Animal Experiment Ethics Committee of Yi Shengyuan Gene Technology (Tianjin) Co., Ltd. (Approval No. YSY‐DWLL‐2024743). Mice received intravenous Ce^3^⁺, Ast (8 mg kg^−1^), CeAst (10 mg kg^−1^), or CeAst@MK (15 mg kg^−1^) 2 h post‐injury and were sacrificed after 24 h for analysis, including renal function (serum BUN, CRE, LDH), inflammatory cytokines (TNF‐α, IL‐1β, IL‐6; ELISA), oxidative stress markers (SOD, GSH, MDA in kidney homogenates), and histopathology (H&E, PAS staining), with body weight and kidney index recorded.

### TUNEL and DHE Staining

Fresh kidney tissues were fixed, embedded in OCT, and cryosectioned (6 µm). Sections were stained with TUNEL or DHE probes following the manufacturer's protocols and counterstained with DAPI. Fluorescence images were acquired using CLSM.

### Immunohistochemistry

KIM‐1 expression and NF‐κB activation were evaluated in paraffin‐embedded renal sections (5 µm) through immunohistochemical staining with specific primary antibodies. Sections were imaged using an Olympus VS200 microscope, and staining intensity was quantified with ImageJ.

### In Vivo Analysis of Macrophage Phenotypes

Kidney sections were stained for CD86 and CD206 and visualized via CLSM. For flow cytometry, kidney tissues were homogenized, RBCs lysed, and cells stained with APC‐CD206, PE‐CD86, and FITC‐F4/80 antibodies. Samples were analyzed by flow cytometry.

### In Vivo Biosafety Evaluation

BALB/c mice were intravenously injected with PBS, CeAst (10 mg kg^−1^), or CeAst@MK (15 mg kg^−1^). After 7 days, blood and major organs were collected for routine blood tests, serum biochemical analysis (ALT, AST, BUN, Cr), and H&E staining.

### Western Blotting

Total proteins from kidney tissues were extracted using RIPA buffer with protease inhibitors, quantified, and separated by SDS‐PAGE. After transfer to PVDF membranes, blots were blocked, incubated overnight with primary antibodies, followed by HRP‐conjugated secondary antibodies. Bands were visualized via ECL and quantified using ImageJ.

### qRT‐PCR

Total RNA from renal tissues was reverse‐transcribed using a one‐step RT‐PCR kit, and quantitative PCR was conducted with SYBR Green Master Mix on an Agilent Mx3000P platform. Each assay was performed in triplicate, with expression levels normalized to housekeeping genes and analyzed by the 2^(−ΔΔCt)^ method. Primer sequences are provided in Table S1 (Supporting Information).

### Network Pharmacology Investigation of Ast for AKI Treatment

This study systematically explored the therapeutic mechanism of Eucommia ulmoides against AKI through an integrated network pharmacology approach. Active components were screened from the TCMSP database using a drug‐likeness (DL) threshold ≥40%. Potential targets were predicted using SwissTargetPrediction and cross‐referenced with AKI‐related targets from GeneCards. PPIs were built using STRING and analyzed in Cytoscape to identify key targets. Functional enrichment analysis (GO terms and KEGG pathways) was conducted with DAVID, and results were visualized using R ggplot2. Molecular docking validation was conducted with AutoDock Vina (v1.2.0), and interaction patterns were visualized using PyMOL (v2.5.4). All analyses adopted a significance threshold of *p* < 0.05.

### RNA Sequencing and Bioinformatics Analysis

Total RNA was extracted from kidneys in AKI and CeAst@MK‐treated groups (*n* = 5). Libraries were constructed and sequenced (Illumina HiSeq 2500, Gene Denovo Biotech). Differential gene expression and pathway enrichment (GO, KEGG, GSEA) were analyzed as described.

### Statistical Analysis

The data were presented as mean ± SD. Statistical comparisons were performed using unpaired Student's *t*‐test or one‐way ANOVA (GraphPad Prism 9). *p* < 0.05 was considered statistically significant. Significance levels were denoted as *p < 0.05, **p < 0.01, ***p < 0.001, and ****p < 0.0001; “ns” indicates no significance.

## Conflict of Interest

The authors declare no conflict of interest.

## Supporting information



Supporting Information

## Data Availability

The data that support the findings of this study are available from the corresponding author upon reasonable request.
