# Supplementary material for: A pH‐Responsive Biomimetic Antioxidant Nanoplatform with Dual Renal Targeting for Synergistic Therapy of Acute Kidney Injury
Source: Adv Sci (Weinh). 2025 Nov 6;13(5):e15664. doi: 10.1002/advs.202515664 (PMC12850223; doi:10.1002/advs.202515664)
Supplement: Supplementary file 1 — Supporting Information [file ADVS-13-e15664-s001.docx]

**A pH-Responsive Biomimetic Antioxidant Nanoplatform with Dual Renal Targeting for Synergistic Therapy of Acute Kidney Injury**

Shichao Zhang^1^, Yuhan Xie^2^, Longchao Zhang^1^, Yuanjiong Qi^1^, Quan Liao^1^, Chenglong Xu^1^, Shushuai Yang ^1^, Qidan Tan^1^, Haiwen Zhou^1^, Shiyong Qi^1*^

1. Department of Urology, Tianjin Institute of Urology, The Second Hospital of Tianjin Medical University, Tianjin 300211, China.
2. Department of Emergency Medicine, Institute of Infectious Diseases, The Second Hospital of Tianjin Medical University, Tianjin, 300211, China.


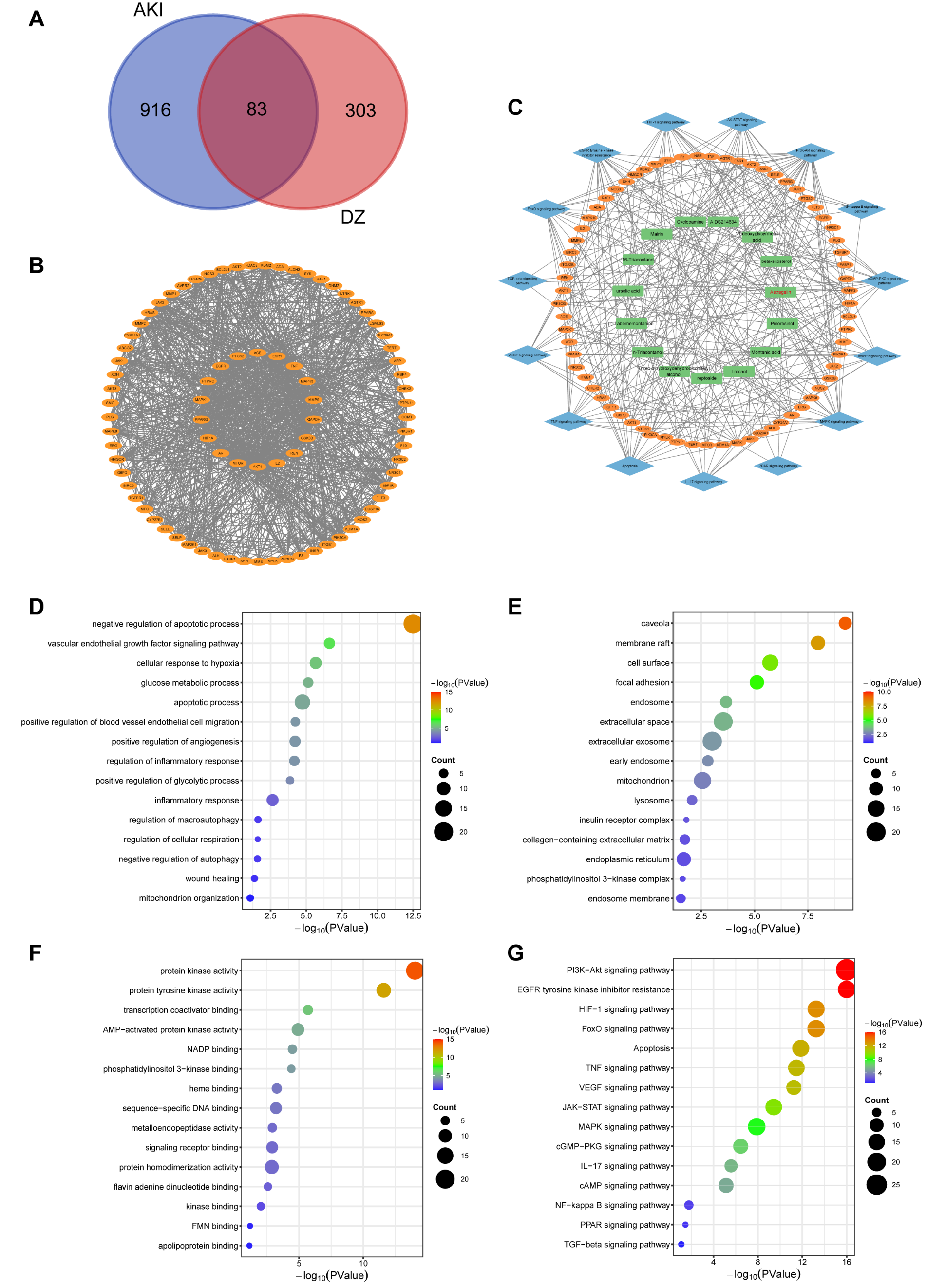


**Figure S1**. Network pharmacological analysis of DZ in the treatment of AKI. (A) Venn diagram of interaction genes between DZ and AKI. (B) PPI network and the 18 hub genes within it. (C) Component-target-pathway networks of DZ and AKI. (D-F) The top 15 entries of GO pathway enrichment analysis. (G) The top 15 entries of KEGG pathway enrichment analysis.


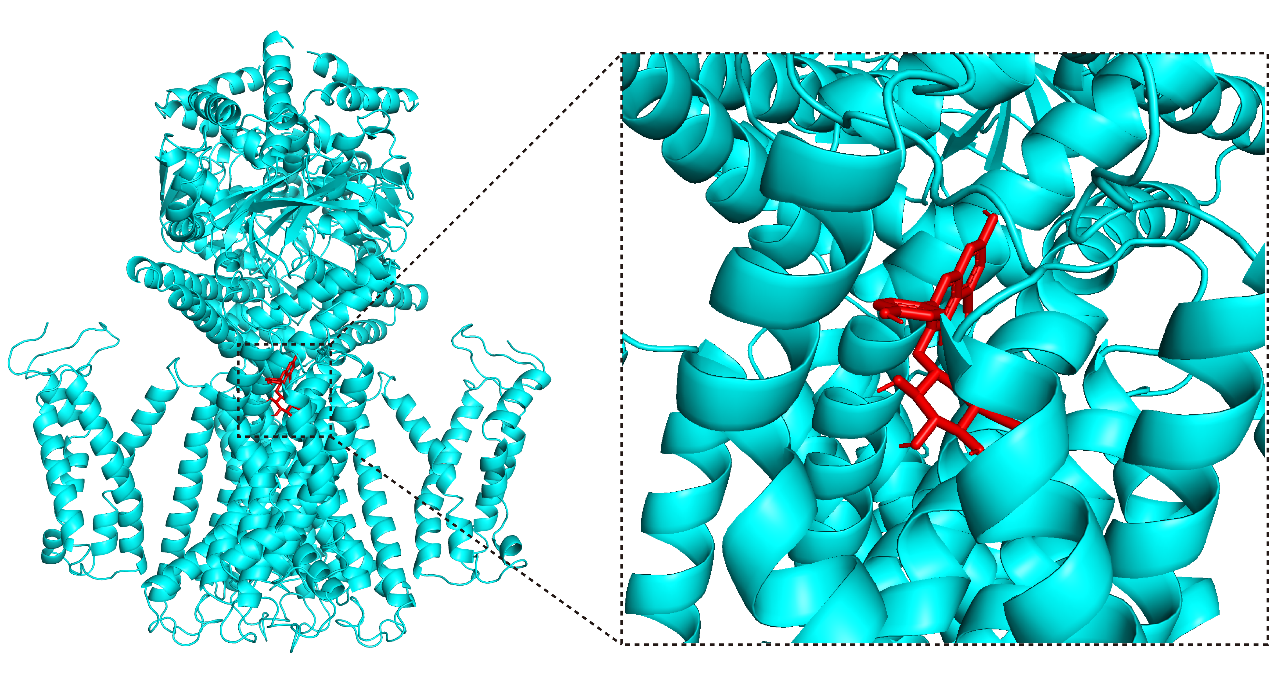


**Figure S2**. Molecular docking of the core therapeutic compound Ast with the core target AKT1.


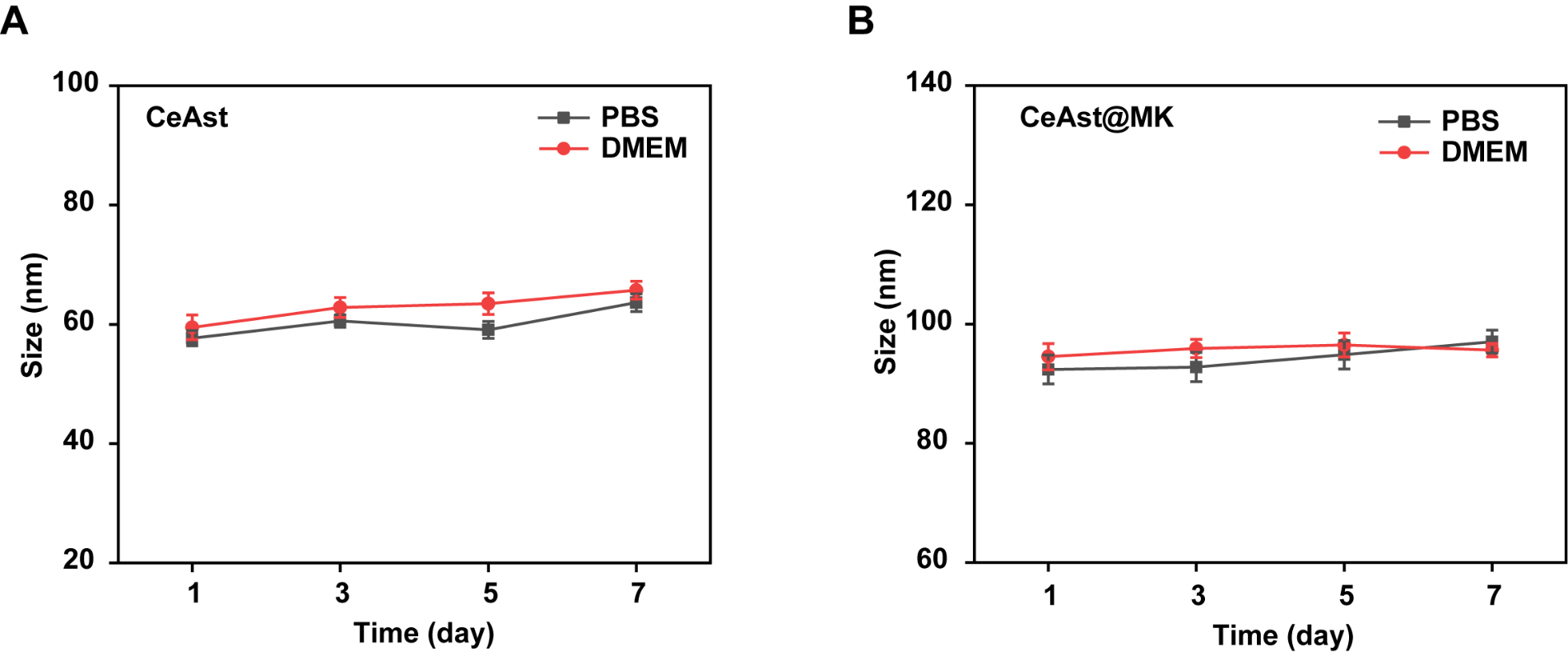


**Figure S3.** Hydrodynamic sizes of CeAst (A) and CeAst@MK (B) in different solutions during storage measured by DLS (n = 3).


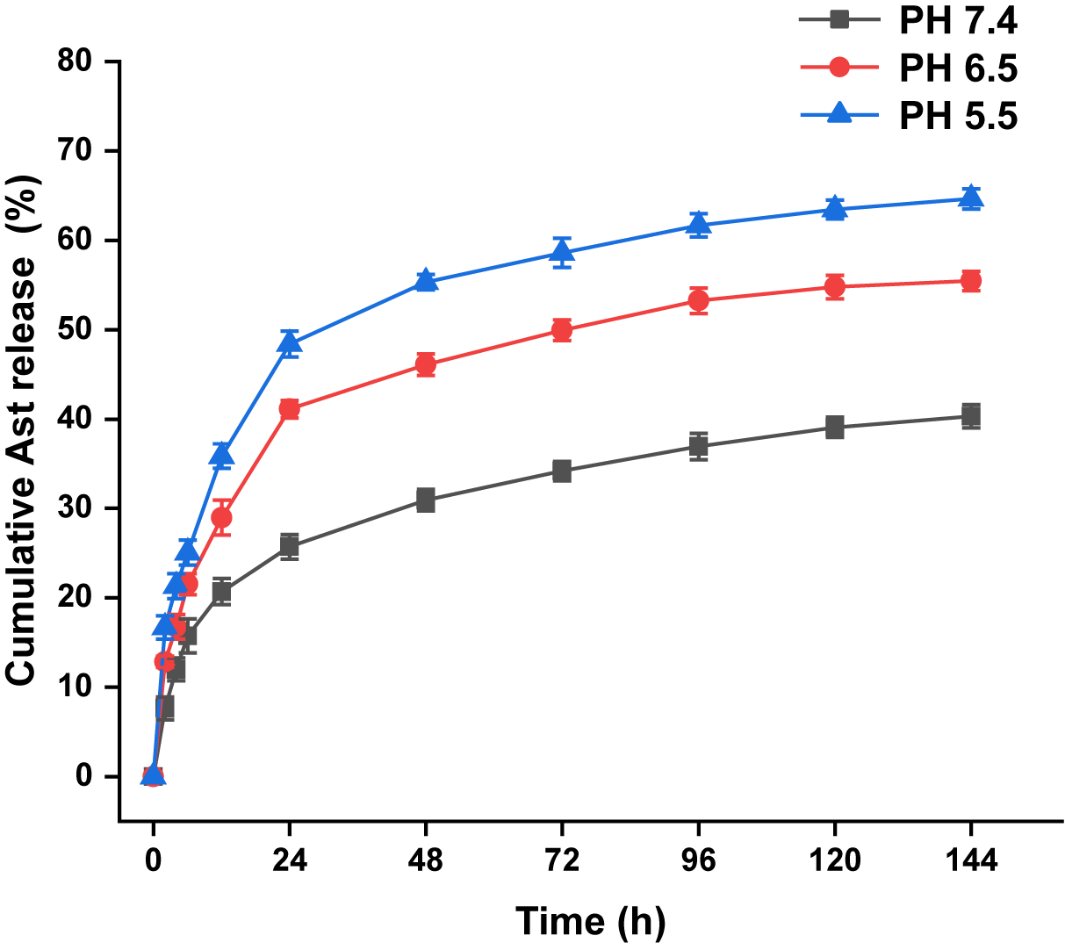


**Figure S4.** Ast release from CeAst@MK under different pH conditions (7.4, 6.5 and 5.5) (n = 3).


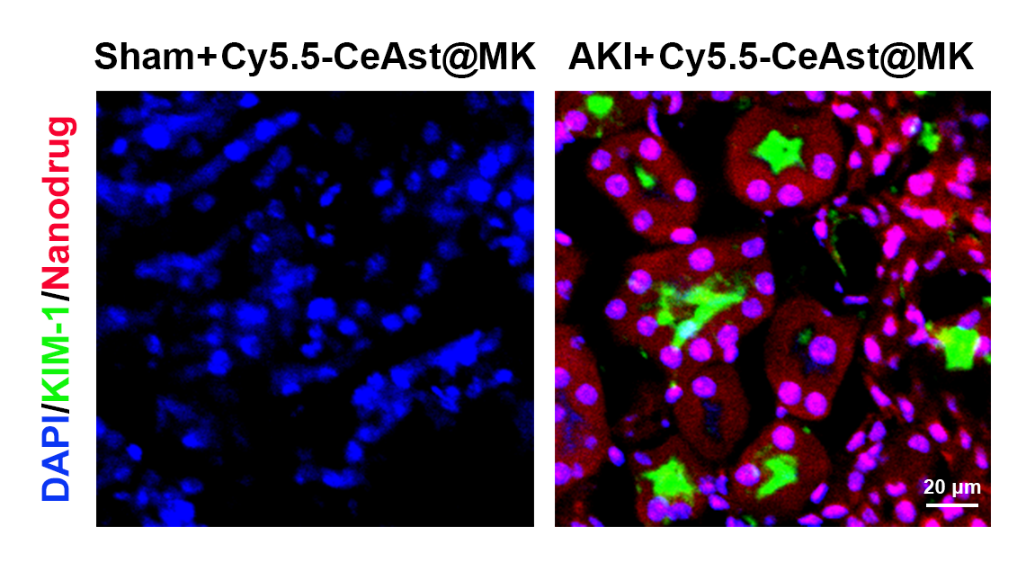


**Figure S5.** Representative IF staining images of Cy5.5-CeAst@MK with KIM-1 markers in Sham and IRI-AKI mice kidney tissues.


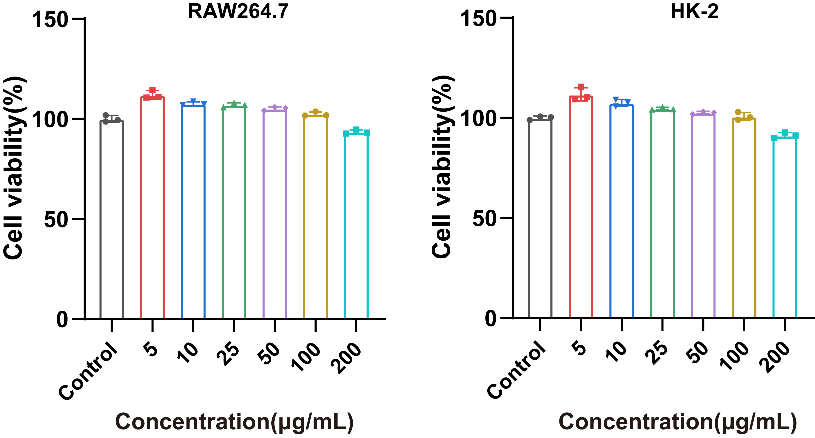


**Figure S6**. The effects of different concentrations of CeAst@MK on the viability of RAW264.7 and HK-2 cells after 24 hours.


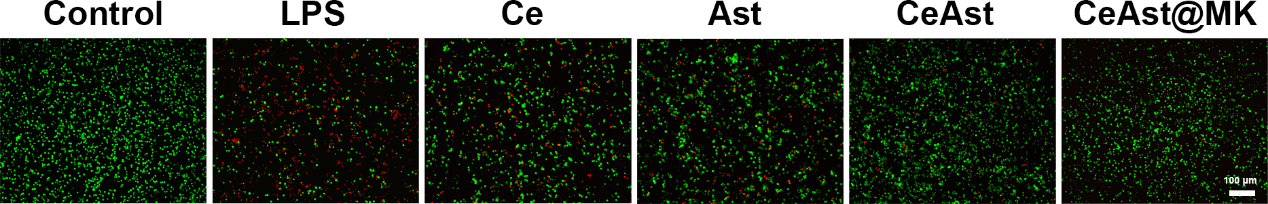


**Figure S7.** Fluorescence images of calcein AM and PI-stained RAW264.7 cells exposed to different treatments.


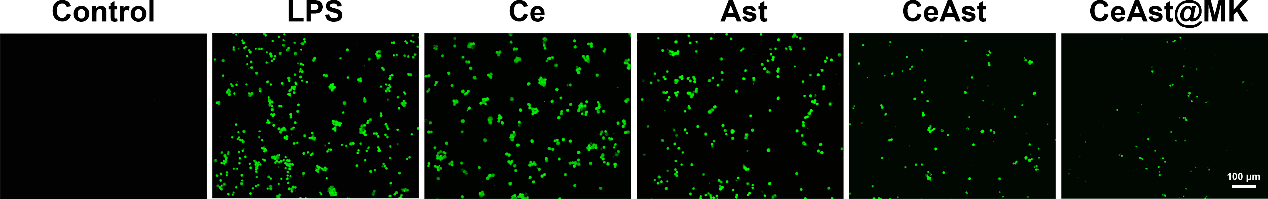


**Figure S8.** Fluorescence images of RAW264.7 cells stained with DCFH-DA under different treatments.


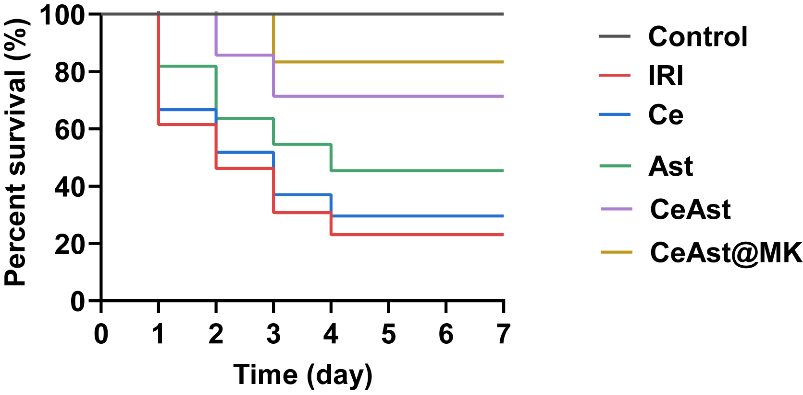


**Figure S9.** Survival rate of mice within 7 days under different treatments (n=6).


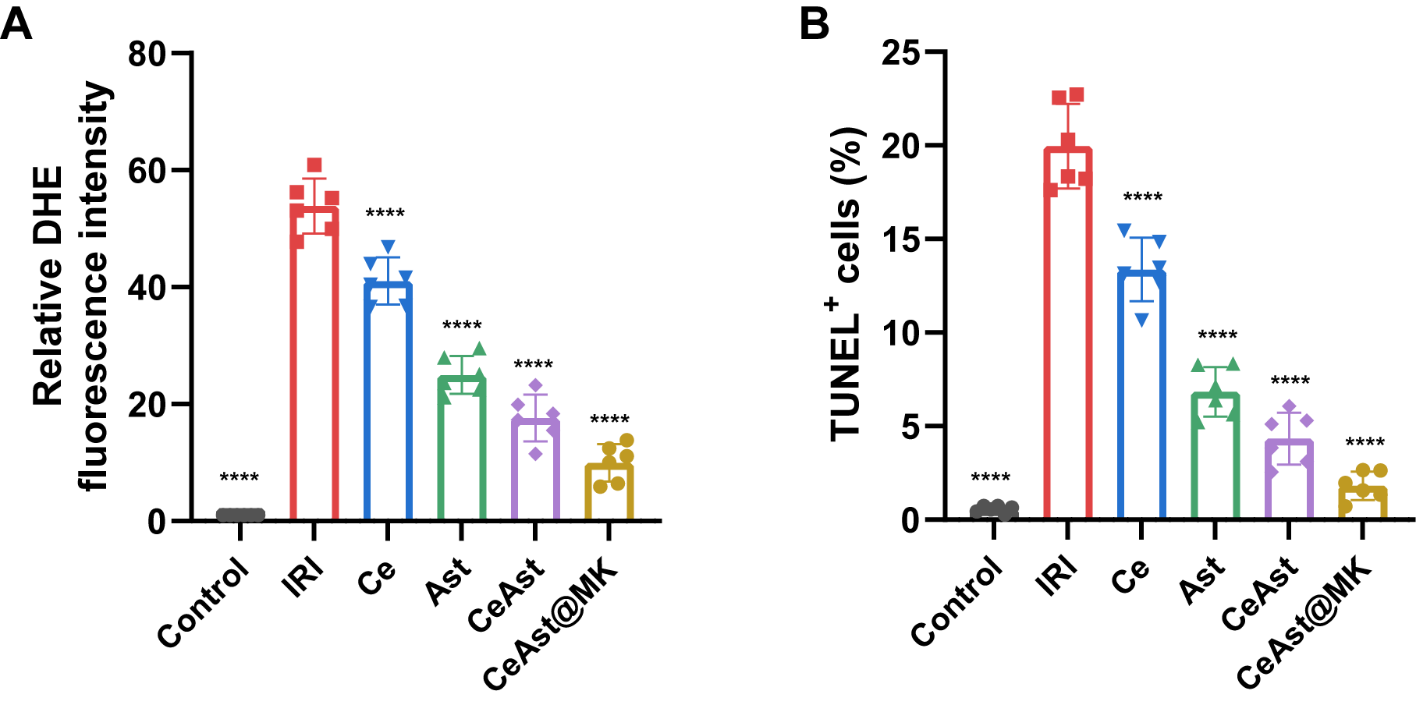


**Figure S10.** Semi-quantitative analysis of DHE (A) and TUNEL (B) fluorescence images in different groups in the IRI-AKI model (n = 6).


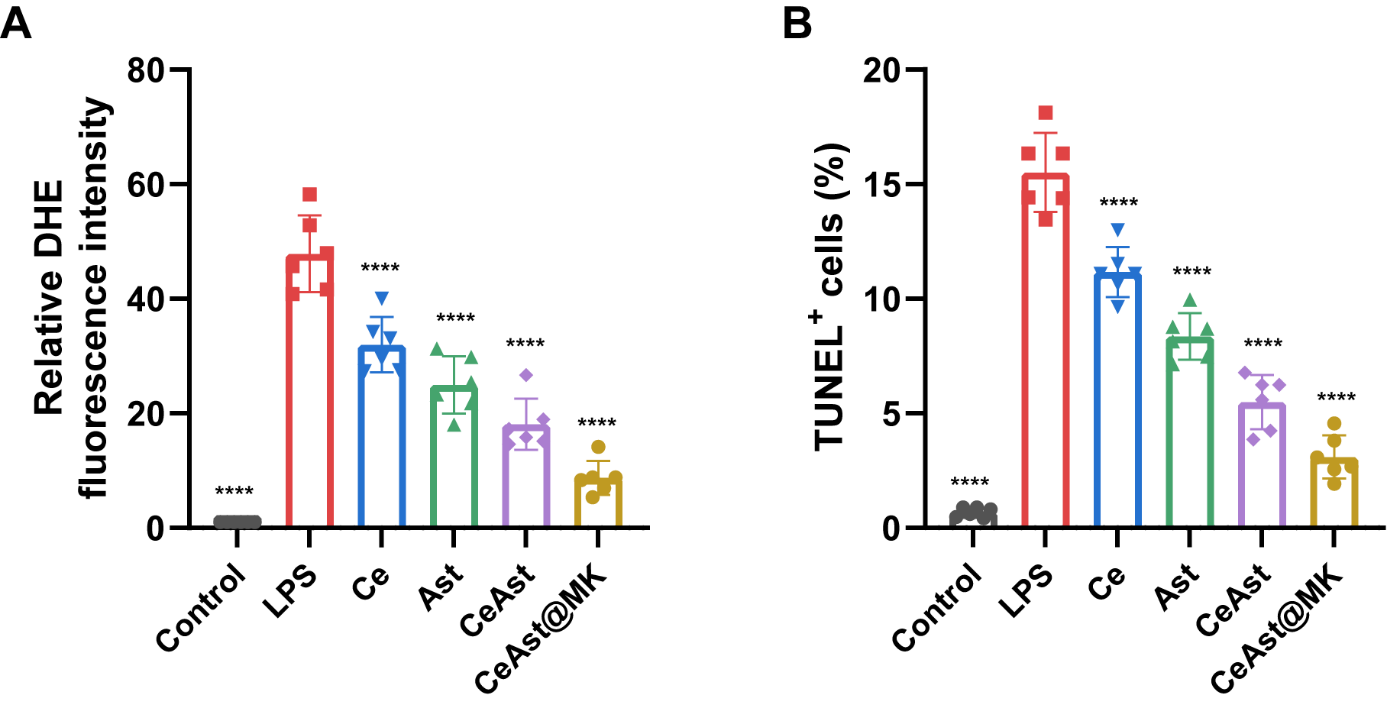


**Figure S11.** Semi-quantitative analysis of DHE (A) and TUNEL (B) fluorescence images in different groups in the LPS-AKI model (n = 6).


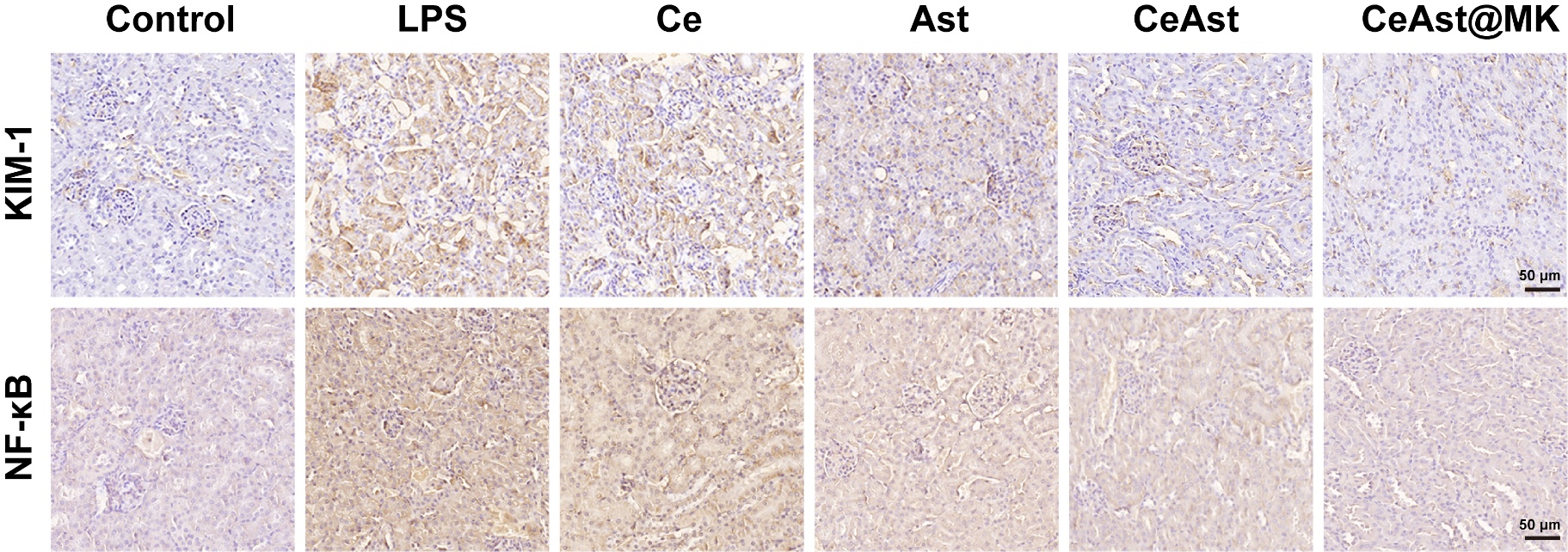


**Figure S12.** Immunohistochemical staining analysis. Immunohistochemical staining results of kidney injury-related biomarkers KIM-1 and NF-κB in kidney tissues from LPS-induced model.


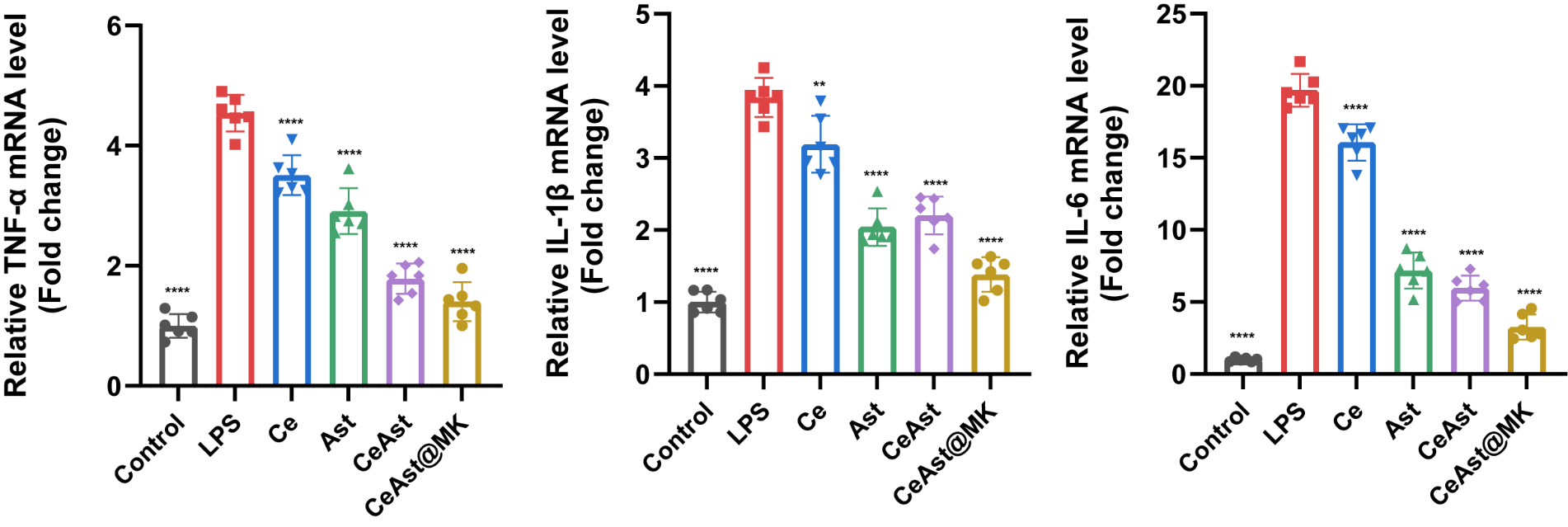


**Figure S13.** The relative renal mRNA levels of inflammatory cytokine genes were measured by real-time quantitative PCR (n = 6).


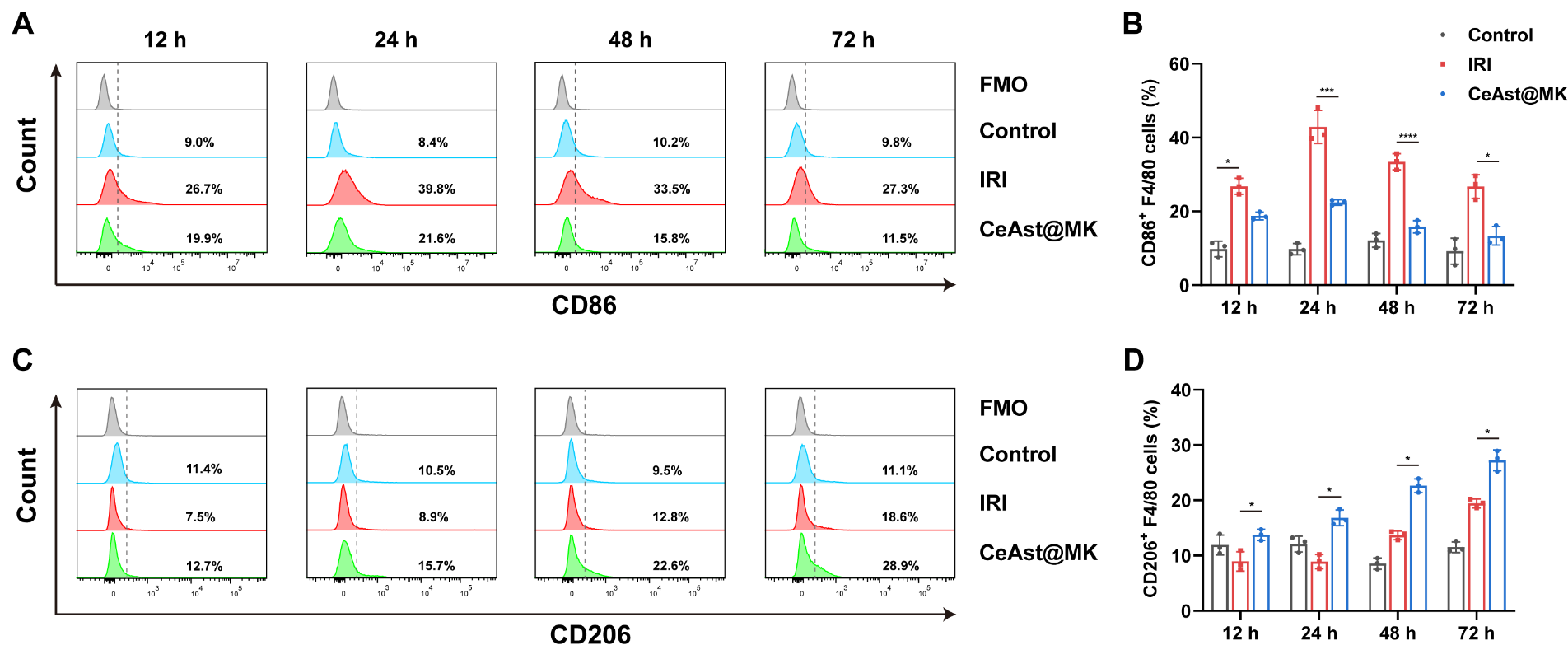


**Figure S14.** Dynamic macrophage polarization in kidney tissues of IRI-AKI mice at different time points (n = 3).


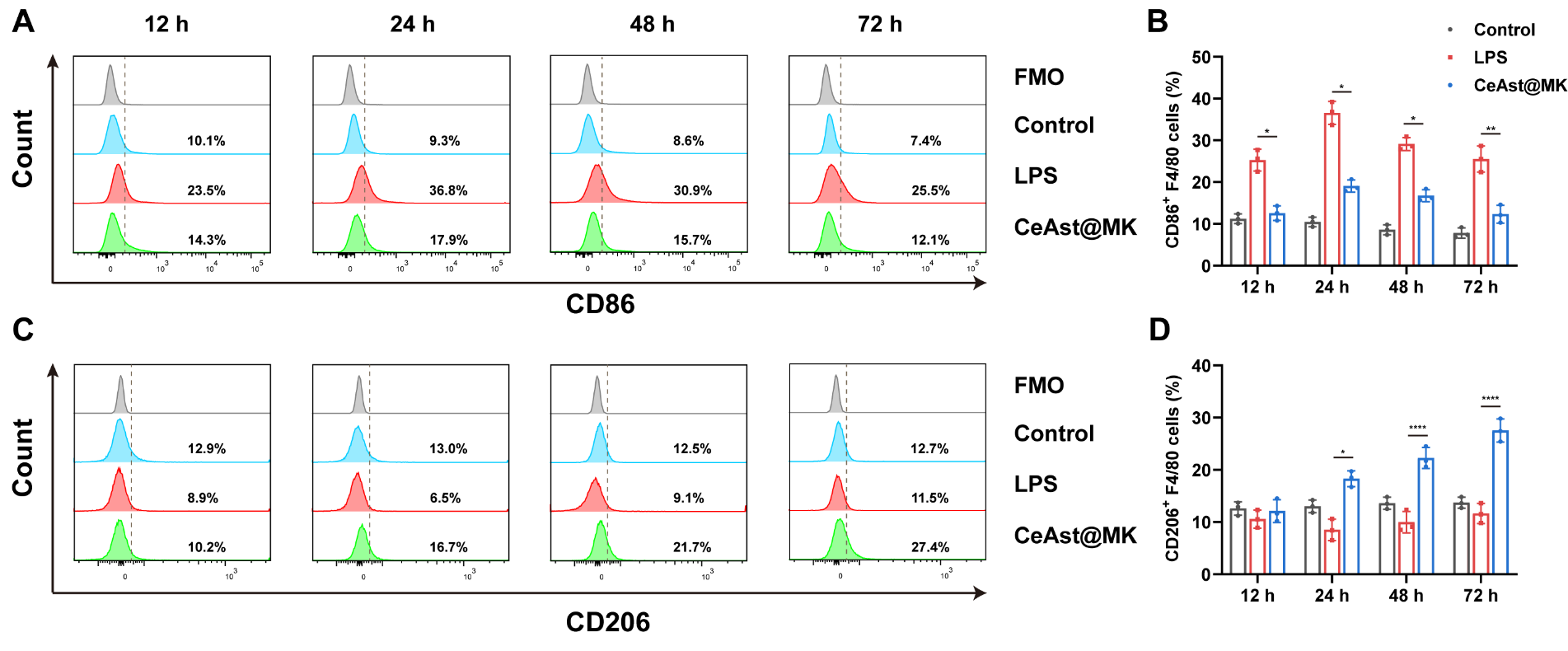


**Figure S15.** Dynamic macrophage polarization in kidney tissues of LPS-AKI mice at different time points (n = 3).


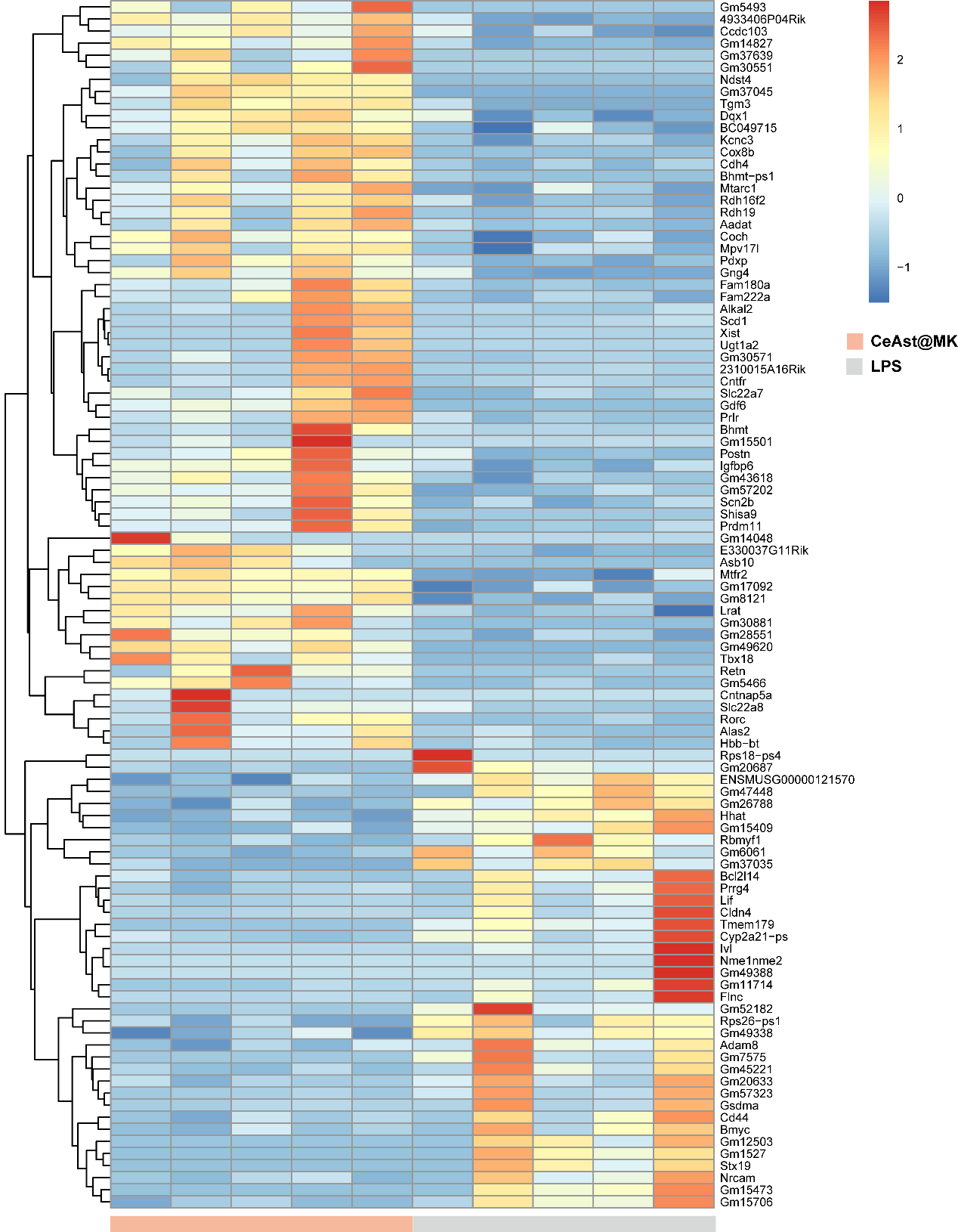


**Figure S16.** Heatmap of the top 100 genes.


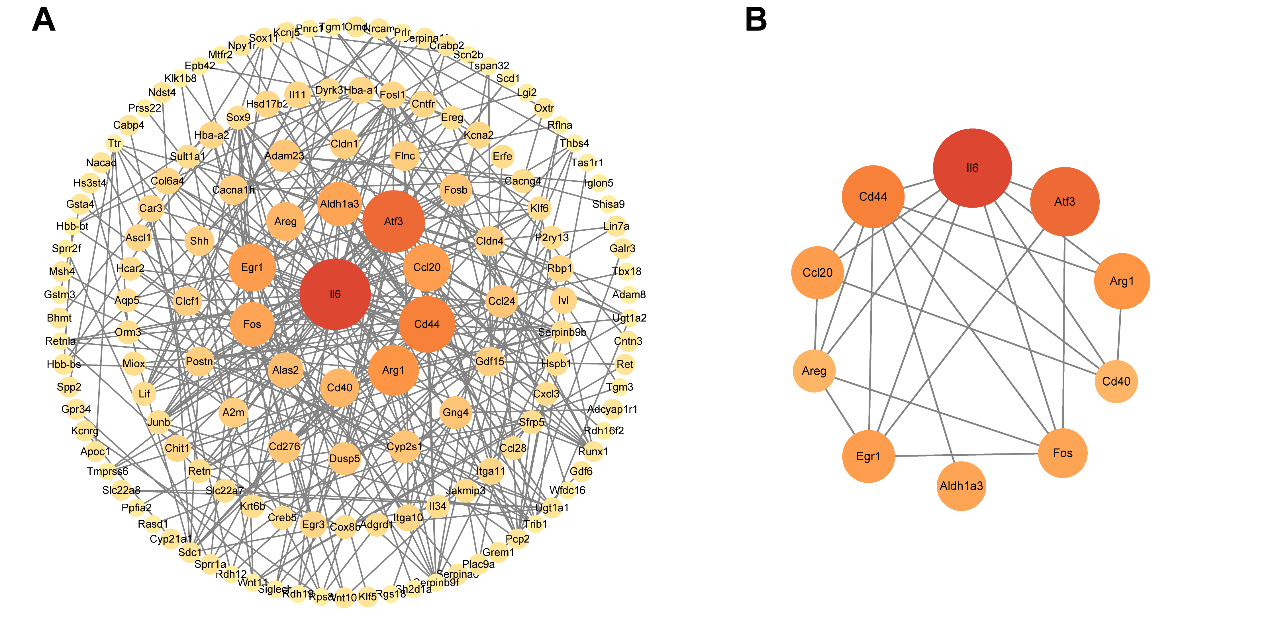


**Figure S17.** Protein-protein interaction network analysis. (A) The network consists of proteins encoded by representative DEGs. (B) The top 10 key nodes ranked in cytoscape and their corresponding interaction network diagram.


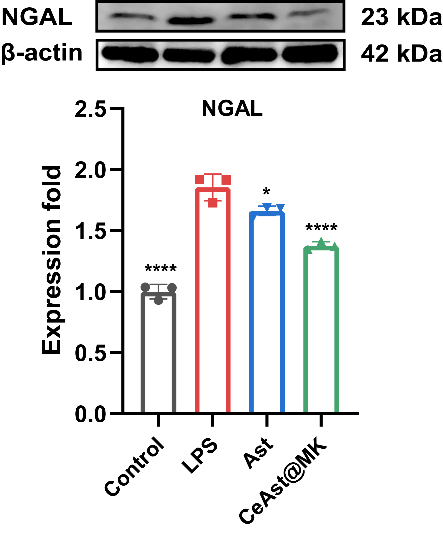


**Figure S18.** Western blot analysis of NGAL expression level and semi-quantitative results. The data are mean ± SD, n = 3.


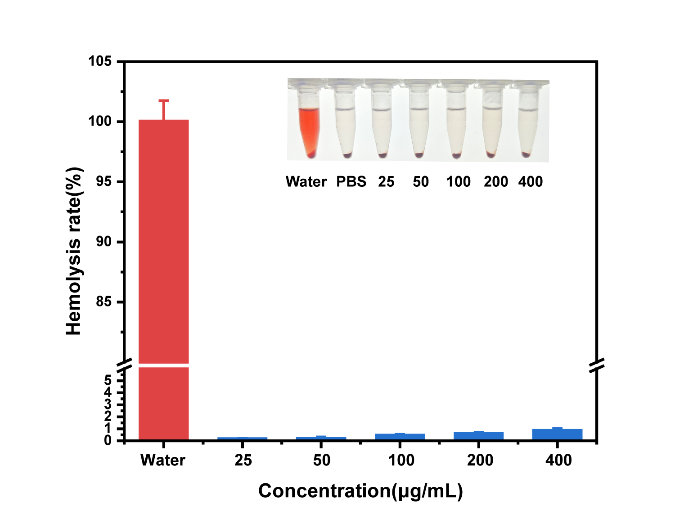


**Figure S19.** Hemolysis test and hemolysis rate results of CeAst@MK.


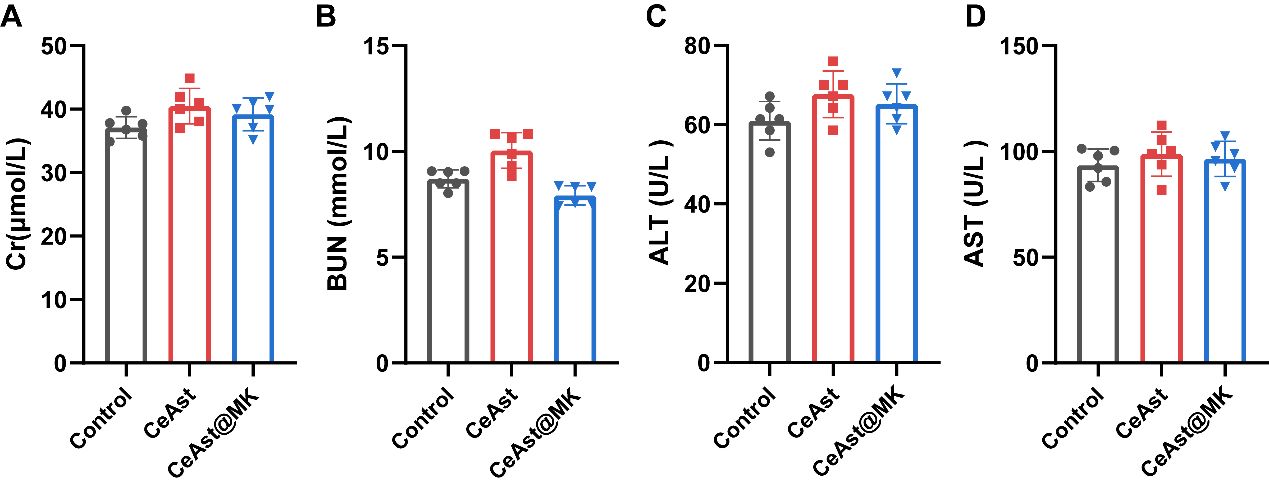


**Figure S20.** (A–D) Changes in serum CRE, BUN, ALT, and AST levels in normal mice with different treatments at 7 days post-intravenous injection.


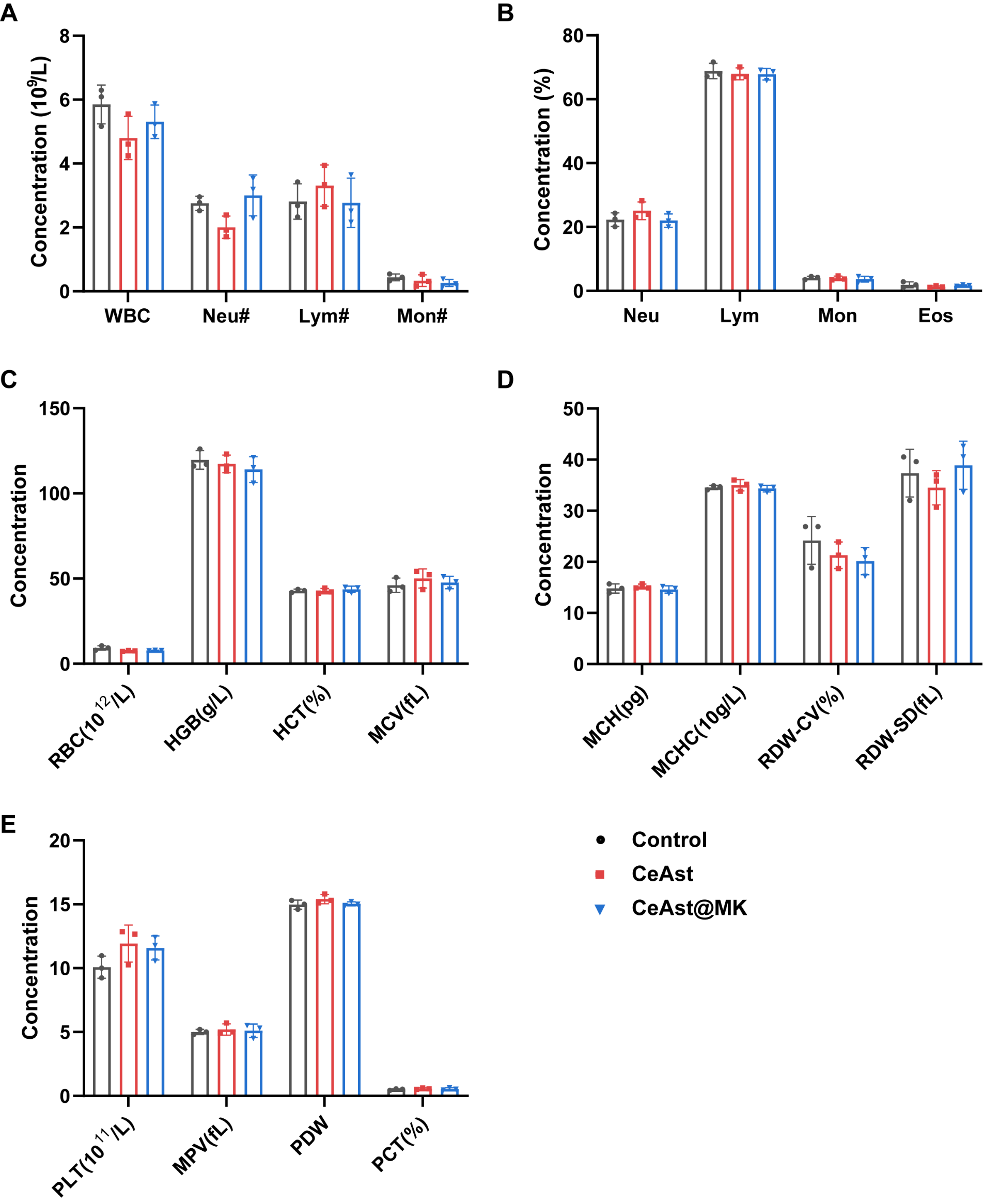


**Figure S21.** (A–E) Routine blood parameters (white blood cells, red blood cells, platelets, etc.) in normal mice with different treatments at 7 days post-intravenous injection.


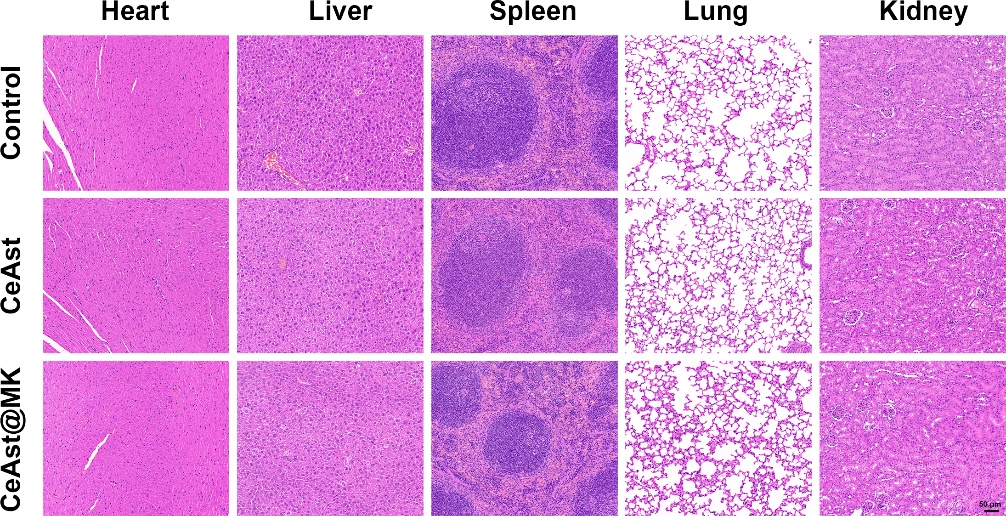


**Figure S22.** HE staining results of heart, liver, spleen and lung tissue sections in normal mice with different treatments at 7 days post-intravenous injection.

**Table S1 The primer sequences**

| Gene | FORWARD (5′–3′) | REVERSE (5′–3′) |
| --- | --- | --- |
| IL-6 | CAACGATGATGCACTTGCAGA | GTGACTCCAGCTTATCTCTTGG |
| TNF-α | GGTGCCTATGTCTCAGCCTCTT | GCCATAGAACTGATGAGAGGGAG |
| IL-1β | GCCACCTTTTGACAGTGATGAG | AAGGTCCACGGGAAAGACAC |
| β-Actin | GGACTGTTACTGAGCTGCGTT | CGCCTTCACCGTTCCAGTT |
